# Genetic diversity and candidate genes for transient waterlogging tolerance in mungbean at the germination and seedling stages

**DOI:** 10.3389/fpls.2024.1297096

**Published:** 2024-03-21

**Authors:** Khin Lay Kyu, Candy M. Taylor, Colin Andrew Douglas, Al Imran Malik, Timothy David Colmer, Kadambot H. M. Siddique, William Erskine

**Affiliations:** ^1^ Centre for Plant Genetics and Breeding (PGB), UWA School of Agriculture and Environment, The University of Western Australia, Perth, WA, Australia; ^2^ The UWA Institute of Agriculture, The University of Western Australia, Crawley, WA, Australia; ^3^ CSIRO Agriculture and Food, Floreat, WA, Australia; ^4^ Department of Agriculture and Fisheries, Gatton Research Facility, Gatton, QLD, Australia; ^5^ International Center for Tropical Agriculture (CIAT-Asia), Lao PDR Office, Vientiane, Lao People’s Democratic Republic

**Keywords:** mungbean mini-core collection, waterlogging, GWAS, tolerance traits, candidate genes, heritability

## Abstract

Mungbean [*Vigna radiata* var. *radiata* (L.) Wilczek] production in Asia is detrimentally affected by transient soil waterlogging caused by unseasonal and increasingly frequent extreme precipitation events. While mungbean exhibits sensitivity to waterlogging, there has been insufficient exploration of germplasm for waterlogging tolerance, as well as limited investigation into the genetic basis for tolerance to identify valuable loci. This research investigated the diversity of transient waterlogging tolerance in a mini−core germplasm collection of mungbean and identified candidate genes for adaptive traits of interest using genome−wide association studies (GWAS) at two critical stages of growth: germination and seedling stage (i.e., once the first trifoliate leaf had fully−expanded). In a temperature−controlled glasshouse, 292 genotypes were screened for tolerance after (i) 4 days of waterlogging followed by 7 days of recovery at the germination stage and (ii) 8 days of waterlogging followed by 7 days of recovery at the seedling stage. Tolerance was measured against drained controls. GWAS was conducted using 3,522 high−quality DArTseq−derived SNPs, revealing five significant associations with five phenotypic traits indicating improved tolerance. Waterlogging tolerance was positively correlated with the formation of adventitious roots and higher dry masses. FGGY carbohydrate kinase domain−containing protein was identified as a candidate gene for adventitious rooting and mRNA-uncharacterized LOC111241851, Caffeoyl-CoA O-methyltransferase At4g26220 and MORC family CW-type zinc finger protein 3 and zinc finger protein 2B genes for shoot, root, and total dry matter production. Moderate to high broad−sense heritability was exhibited for all phenotypic traits, including seed emergence (81%), adventitious rooting (56%), shoot dry mass (81%), root dry mass (79%) and SPAD chlorophyll content (70%). The heritability estimates, marker−trait associations, and identification of sources of waterlogging tolerant germplasm from this study demonstrate high potential for marker−assisted selection of tolerance traits to accelerate breeding of climate−resilient mungbean varieties.

## Introduction

Mungbean (*Vigna radiata* (L.) R. Wilczek var. *radiata*) is a major subtropical short−season pulse crop in Asia and other parts of the world. The species is native to the Indo−Burma region (Jain and Mehra, 1978) where it is widely cultivated, with India and Myanmar each supplying about 30% of annual global production, followed by China (16%) and Indonesia (5%) (Nair and Schreinemachers, 2020). Within these countries, mungbean is a vital source of nutritional food for poor and anaemic women and children (Nair et al., 2012). Additionally, the crop is of tremendous importance to smallholder farming families due to the income it provides as a cash crop and the agronomic value it provides to cropping systems. For example, mungbean can increase the productivity of subsequent rice crops by up to 8% because it fixes soil nitrogen through symbiosis and helps to break pest and disease cycles (Weinberger, 2003). Due to these combined benefits, mungbean is widely grown in both upland and lowland farming systems in Asia (Islam et al., 1993; Herridge et al., 2019). In upland ecosystems of Southeast and South Asia, mungbean is grown as an intercrop with other legumes, such as pigeonpea (*Cajanus cajan* L.), oilseeds [sesame (*Sesamum indicum* L.) or groundnut (*Arachis hypogaea* L.)] or cereal crops [sorghum (*Sorghum bicolor* L.) and maize (*Zea mays* L.)] in pre-monsoon and monsoon seasons (Islam et al., 1993; Herridge et al., 2019). In lowland ecosystems, mungbean is adopted as a relay crop, broadcast onto the standing rice crop 7–10 days before harvest or dibbled manually after harvest (Gupta et al., 2016).

The global area sown to mungbean has increased from 4.6 to 7.3 million ha, with production and productivity increasing from 2.3 to 5.3 million tonnes and 500 to 721 kg ha^–1^ (Hartman et al., 1993; Nair and Schreinemachers, 2020). However, it is yet to reach its genetic yield potential (Douglas et al., 2020), with abiotic stresses caused by extreme weather events challenging crop productivity (Oshunsanya et al., 2019). Increased waterlogging has devastated crop production in some parts of the world and is becoming increasingly problematic due to a high frequency of unseasonal rainfall events (IPCC, 2021). For example, 69−year average annual weather data from Barisal, Bangladesh, indicates that the cumulative frequency of heavy rainfall is challenging farmers to find an optimum sowing time for mungbean after a T-Aman rice crop (unpublished ACIAR annual finding, June 2018). The Food and Agricultural Organisation estimates that waterlogging, as a result of high frequency of unseasonal rainfall, has been found to be destructive to agricultural production, based on the findings of 78 natural disasters in 48 regions (Food and Agriculture Organization of the United Nations, 2015). Floods and soil waterlogging significantly contributed to a reduction of $21 billion in crop and livestock losses between 2008 and 2018 (FAO, 2021). Thus, increased occurrences of soil waterlogging have become a major abiotic stress associated with global climate change.

Waterlogging can occur at any stage of plant development in rainfed and irrigated cropping systems due to irregular rainfall patterns (Setter and Waters, 2003). However, excess soil water immediately before or after rice harvest makes germination a particularly vulnerable developmental stage for the succeeding relay crops, and poor crop establishment is documented in both field and glasshouse experiments for different legumes in such conditions (Zaman et al., 2018). For examples, this type of waterlogging stress has been observed in the following legume relay crops grown after rice, including field pea (*Pisum sativum* L.), lentil (*Lens culinaris* L.), grass pea (*Lathyrus sativus* L.), and soybean (*Glycine max* L.), in countries in South, Southeast and East Asia, including Bangladesh, India, Nepal, Pakistan and Japan (Araki, 2006; Malik et al., 2015; Zaman et al., 2018). Established crops also face critical challenges. Islam et al. (2008) reported that waterlogging of mungbean crops damages roots, reduces leaf chlorophyll content, and ultimately decreases grain yield. In addition, the oxygen deprivation in the rhizosphere due to transient soil waterlogging reduces the N_2_ fixation activity of root nodules in mungbean (Singh and Singh, 2011; Kyu et al., 2021) and soybean (Suematsu et al., 2017), which limits the capacity of the crops to provide soil improvement benefits. This ultimately compromises one of the important sustainable agricultural benefits of growing mungbean and other legume crops. Unfortunately, the increased frequency and severity of extreme weather events, such as flooding, due to climate change mean that mungbean crops will be more likely to experience transient waterlogging in lowland systems in future, which will greatly reduce production (Rötter et al., 2012; Rötter et al., 2013).

One solution to mitigate the detrimental impacts of excessive soil moisture in Asia is to breed new varieties of mungbean with genetic tolerance to transient waterlogging stress. Successfully accomplishing this, however, firstly requires a solid understanding of (i) how much phenotypic variation exists for transient waterlogging stress tolerance, (ii) the strategies tolerant mungbean plants use in response to stress, and (iii) the genetic control of these adaptive responses. The degree of tolerance to waterlogging in plants depends on the stage of growth (VanToai et al., 1994), plant growth habit, and the duration of stress (Greenway et al., 1994). Mungbean is susceptible to transient soil waterlogging throughout its life cycle (Nair et al., 2012), but is especially vulnerable during early growth (Tickoo et al., 2006; Douglas et al., 2020). Some varietal differences in mungbean response to transient waterlogging at the late vegetative stage have been reported (Bagga et al., 1984; Ahmed et al., 2002; Islam et al., 2007, 2008). However, characterisation of broader phenotypic diversity in transient waterlogging tolerance in mungbean has not been conducted, nor has exploration of the genetic basis for tolerance. Systematic screening of a wide range of germplasm is therefore needed to identify genetic diversity and genetic loci, particularly during early development when crops are most susceptible.

Genetic variation for waterlogging tolerance has been studied in other legume crops, including soybean (Shannon et al., 2005; Suematsu et al., 2017), common bean (*Phaseolus vulgaris* L.) (Soltani et al., 2017) and pigeonpea (Krishnamurthy et al., 2012; Sultana et al., 2012) using large sets of germplasm from different origins, including core and mini−core collections. Such collections contain genetically diverse germplasm that can be used for comprehensive studies of intraspecific variation using genome−wide association studies (GWAS) and can be subsequently exploited to develop climate−resilient genotypes with biotic and abiotic stress tolerance (Upadhyaya et al., 2008). GWAS has superior resolution mapping power, using mass recombination events from numerous meiotic events throughout the germplasm’s evolutionary history to characterise several alleles concurrently in diploid (Zhao et al., 2007) and polyploid (Breseghello and Sorrells, 2006) crops.

Mungbean is an orphan crop; however, recent efforts have developed genetic and genomic resources for whole−genome scan studies, such as GWAS. Kang et al. (2014) constructed the first draft genome sequence of mungbean to facilitate genomic research. In addition, the World Vegetable Center (WorldVeg) has created a mungbean mini−core germplasm collection representing genetic resources from more than 6,700 accessions based on genotypic and phenotypic traits (Schafleitner et al., 2015). From this mini−core collection, useful lines and candidate genes have been identified for numerous traits, such as hypocotyl pigmentation and maturation under abnormally hot weather and different photoperiods (Sokolkova et al., 2020), seed coat lustre (Breria et al., 2019), salinity tolerance at germination (Breria et al., 2020), seed size (Akhtar et al., 2021) and favourable root traits for heat and drought stress resistance (Aski et al., 2021). However, transient waterlogging tolerance is notably absent from this list.

We hypothesised that the mungbean mini−core collection contains functional genetic variation for transient waterlogging tolerance. To this end, we conducted the first screen of the WorldVeg mungbean mini−core collection for genotypic variation in response to transient waterlogging stress at the germination and seedling stages with the aim of identifying sources of tolerant germplasm and as a precursor to better understanding the genetic regulation of tolerance in this legume.

## Materials and methods

### Plant material

A set of 292 mini−core collection mungbean genotypes from nine regions was assayed separately for transient waterlogging response at two critical stages of growth: germination and seedling stages. The mini−core collection was developed by WorldVeg (Schafleitner et al., 2015) (**Supplementary Table 1**) and seed was obtained from The Department of Agriculture and Fisheries, Queensland. For practicality, the genotypes were randomly assigned to two cohorts for germination screening and three cohorts for seedling stage screening. Eight check genotypes (BARIM−3, BARIM−6, Celera II−AU, Jade−AU, VI 2173, VI 2537, VI 4069, VI 4954) were replicated five times in each cohort at the germination stage, and once in each cohort at the seedling stage due to limited space. The selection of these check genotypes was based on criteria focusing on emergence, adventitious root formation, and the maintenance of shoot and root growth during and after waterlogging treatment.

### Methods

#### Experimental conditions

The experiments were conducted in a temperature−controlled glasshouse at The University of Western Australia (UWA), Crawley, Western Australia (31° 59’ S, 115° 49’ E) from May to June 2019 (germination stage) and September to December 2019 (seedling stage). The temperature in the glasshouse ranged from 21 ± 4°C (night) to 32 ± 3°C (day) with 10 h 45 min of daylight (1,150–1,627 µmol m^–2^ s^–1^) on average. The seeds were sown in the same red−brown sandy clay loam (Calcic Haploxeralf) used for waterlogging studies in pea (*Pisum sativum* L.) (Zaman et al., 2018), grass pea (*Lathyrus sativus* L.) (Wiraguna et al., 2020) and mungbean and blackgram (*Vigna mungo* L.) (Kyu et al., 2021), which was oven−dried before sieving in a 2 mm soil sieving machine.

#### Experimental design

All experiments were in a split−plot design with three replications. Within replicates a spatial row–column blocking design was used to control variation to improve the precision of treatment comparisons. Stress treatment (waterlogging vs. drained control) was the main plot factor with genotype in the sub−plots. A pot was considered an experimental unit. The optimal transient soil waterlogging duration to identify variation in waterlogging response for the main factor was four days at the germination stage and eight days at the seedling stage (Kyu et al., 2021).

#### Germination stage screening

The screening procedures for waterlogging and data recording were consistent with our previous findings (Kyu et al., 2021). Briefly, before sowing, seeds were surface−sterilised with 1% commercial bleach for 1 min and rinsed with deionised water four times. P−Pickel T liquid fungicide [Thiram (360 g L^–1^) + Thiabendazole (200 g L^–1^)] was applied at 300 mL 100 kg^–1^ seed. Twenty seeds of each genotype were sown per pot at 10 mm depth and covered with soil. Pots were 0.8 L (90 × 90 × 180 mm) with drainage holes (~10 mm) in the base, covered with filter paper to avoid soil loss prior to filling the pots with 100 g gravel, followed by 1 kg soil. Following potting, all pots were placed in 60 L plastic tanks and kept at 80% field capacity for 2 days before sowing. Twelve platinum electrodes—six each for drained and waterlogged pots—were placed at 100 mm depth to monitor soil redox potential (Zaman et al., 2018).

Immediately after sowing, the waterlogging treatment was imposed by adding deionised (DI) water to fill the 60 L tanks up to the level of the soil surface. The soil water table was retained at the soil surface level throughout the waterlogging treatment by adding additional DI water to the tanks as required. Drained control pots were kept at 80% field capacity by adding DI water directly to the pots as required. After 4 days of waterlogging, treated pots were relocated into free−draining plastic tanks to record emergence and seedling growth during recovery. The 80% field capacity was maintained in the recovery pots by adding additional DI water as required.

Soil redox potential was measured daily with platinum electrodes (Pt) and an Ag–AgCl reference electrode using a handheld Digital Multimeter (Fluke 114, Everett, Washington, USA). Redox values were calculated according to the method by Patrick et al. (1996). After 7 days of recovery, germinated seedlings were gently washed from the soil with tap water for measurements. Waterlogged and drained control pots were harvested on the same day.

At harvest, seedlings with at least one trifoliate leaf were counted as fully grown emerged plants. After plant washing, harvested plants were separated into shoots and roots and oven−dried at 60 °C for 3 days to measure dry mass.

#### Seedling stage screening

For seedling screening, the experimental pots were free−draining 4 L plastic pots (145 × 145 × 220 mm). Drainage holes (15 mm) were covered with filter paper before filling with 500 g gravel followed by 4 kg sieved dry soil. The pots were placed in 60 L plastic tanks (10 per tank) and retained at 80% field capacity two days prior to sowing. Each pot received 40 mg kg^–1^ dihydrogen ammonium phosphate [(NH_4_) (H_2_PO_4_)] based on the soil analysis (**Supplementary Table 2**). The sterilised seeds were placed on 55 mm diameter Grade 1 Whatman filter paper in a 55 mm diameter Petri dish and incubated overnight in a temperature−controlled room (25°C). The next day, six germinated seeds were placed at 30 mm depth. The seeds were inoculated with Group I mungbean Rhizobium strain CB 1015 (New Edge Microbial, New South Wales, Australia) and then covered with 2 mm sieved dry soil. Each pot was thinned to two seedlings with similar vigour 4 days after emergence. Waterlogging was imposed 15 days after sowing (DAS), when the first trifoliate leaf had opened fully, maintaining the soil water table 10 mm above the topsoil surface. The drained controlled pots were maintained at 80% field water capacity. Twelve Pt electrodes—six in waterlogged and six in drained control pots—were placed at 100 mm depth in randomly selected pots of each waterlogged and drained soil to measure soil redox potential. After 8 days of waterlogging, the waterlogged pots were drained to observe plant growth during 7 days of recovery, with the experiment terminating at 30 DAS.

SPAD chlorophyll content was measured immediately after the waterlogging period on the first trifoliate leaves (23 DAS) of waterlogged and drained control plants with a handheld Minolta SPAD 502 (Konica−Minolta, Japan). At harvest, the soil was removed from the roots of each plant with tap water before phenotyping for four agronomic traits: shoot, root and total dry mass and adventitious root formation. The number of adventitious roots was counted. Shoots and roots were separated, oven−dried at 60°C for 3 days and then weighed.

### Statistical analysis of phenotypic data

Analyses of variance (ANOVA) of check genotypes and mini−core collection genotypes were undertaken in a split−split plot design to understand the homogeneity among cohorts and the effect of different cohorts on check genotypes, treatments, and their interactions. Further, best linear unbiased predictors were determined for block effects using Genstat 21^st^ edition (VSN International, UK). Tables were constructed using spatial analysis of row–column design according to linear mixed models (REML: restricted maximum likelihood) at each growth stage. The predicted means of REML were analysed to estimate genetic diversity parameters: mean, minimum, maximum, standard deviation, skewness, and kurtosis. Variance components due to genotype (σ^2^_g_), error (σ^2^_e_) and broad−sense heritability (H^2^) for each trait were estimated with RStudio 4.1 (Falconer and Mackay, 2005; Schmidt et al., 2019). A frequency distribution of variation for the traits of interest (expressed as % of control) was undertaken. A one−way ANOVA was performed to assess the relationship between geographic region of origin and phenotypic responses. Pearson’s correlation, regression, and principal component analyses (PCA) of quantitative trait data were carried out in GENSTAT 21^st^ edition to explore the relationships between variables under waterlogging and control conditions.

### Genomic by sequencing

Identification of Single nucleotide polymorphism (SNP) markers for the mungbean mini-core collection was done by Diversity Array Technology (DArT) P/L, Australia (http://www.diversityarrays.com) using the mungbean genome sequence, Vardi_ver6 (Kang et al., 2014) as a reference, resulting in 24,870 raw SNPs.

### SNP marker filtering and genome-wide linkage decay

The raw SNP dataset was subjected to stringent quality filtering using the dartR v2.7.2 package (Gruber et al., 2018; Mijangos et al., 2022) in R v4.2.2 software (R Core Team, 2022). Loci that did not map to physical locations on chromosomes within the mungbean reference genome (6,330 SNPs) were excluded from further analyses. In addition, 15,018 SNPs with monomorphic loci, call rates of <0.95 and minor allele frequency (MAF) of <0.05 were removed. A total of 3,522 high−quality SNPs remained for genetic analyses after filtering (**Supplementary Table 3**). The filtered SNPs were distributed unevenly across the 11 mungbean chromosomes (**Supplementary Table 4**), with an average density per chromosome of one SNP per 91.5 Kb. The filtered SNPs had an average heterozygosity proportion of 0.22. The pairwise linkage disequilibrium (LD) between the 3,522 high-quality SNPs was estimated by allele frequency correlations (r^2^) using TASSEL software (V5.1.0) (Bradbury et al., 2007; Tao et al., 2013). LD decay was estimated by fitting a smooth spline of averaged r^2^ over physical distance in R software. The LD decay distance was calculated at the distance where the average r^2^ decreased to half its maximum value (Hill and Weir, 1988; Remington et al., 2001).

### Population structure analysis

The population structure of the mini−core genotypes was analysed using the filtered SNP set in STRUCTURE 2.3.4 (Pritchard et al., 2010). STRUCTURE performs a Bayesian model−based clustering approach applying Markov Chain Monte Carlo (MCMC) estimation (Porras-Hurtado et al., 2013). Nine K values (K=2:10) with five replicated runs each were analysed. The burn−in period was set at 5,000 with 10,000 MCMC replications. The admixture model was chosen as the ancestry model assumption. The subpopulation (K) values obtained from STRUCTURE were mined by STRUCTURE Harvester (Earl and von Holdt, 2011). The most likely structure value was determined using the non−parametric Wilcoxon test. A threshold of non−admixed individuals was set at a Q matrix value ≥70%. Admixed individuals were classified as having Q matrix values <70%.

Principal Component Analysis (PCA) was conducted using the dartR package in R software. PCs that individually accounted for >10% of the total observed variation were plotted to reveal population structure.

Finally, the evolutionary history of the mini−core was inferred using the neighbour−joining method (Saitou and Nei, 1987). A bootstrap consensus phylogenetic tree inferred from 100 replicates (Felsenstein, 1985a) was chosen to best represent the evolutionary history of the taxa analysed (Felsenstein, 1985b).The optimal tree is drawn to scale, with branch lengths in the same units as those of the evolutionary distances used to infer the phylogenetic tree. The evolutionary distances were computed using the Maximum Composite Likelihood method (Tamura et al., 2004), with the number of base substitutions per site as units. The analysis involved 292 nucleotide sequences. All ambiguous positions were removed for each sequence pair (pairwise deletion option) of the final data. Evolutionary analyses were conducted in MEGA11 (Stecher et al., 2020; Tamura et al., 2021).

### Genome-wide association studies and candidate gene prediction

GWAS analyses were undertaken with the R package Genomic Association and Prediction Integrated Tool, GAPIT version 3 (Lipka et al., 2012; Wang and Zhang, 2021). The analyses were performed using 3,522 high−quality filtered SNP markers and phenotypic data expressed as a reduction rate (i.e. % of control) for emergence, shoot, root, and total dry mass, and SPAD chlorophyll content. The reduction rate transformation of phenotype data was necessary so that genetic responses to transient waterlogging stress could be analysed, rather than genetic variation underlying inherent agronomic performance, which would be detected using the original phenotypes under waterlogged and controlled conditions. Unadjusted phenotype data were used for adventitious root formation, however, as there were no adventitious roots in their drained−controlled pots.

Multiple statistical models were tested per trait, including: (i) general linear model (GLM, Price et al., 2006), (ii) mixed linear model (MLM, Yu et al., 2006), (iii) compression MLM (CMLM, Zhang et al., 2010), (iv) fixed and random model circulating probability unification (FarmCPU; Liu et al., 2016) and (v) Bayesian−information and Linkage−disequilibrium Iteratively Nested Keyway (BLINK; Huang et al., 2019). The most appropriate GWAS statistical model was identified by inspecting the quantile−quantile (Q−Q plots) and Manhattan plots. BLINK was deemed the most reliable of the five statistical models tested as it had the least evidence of *P*−value inflation and was therefore selected for use in the final analyses.

**Figure 1:**
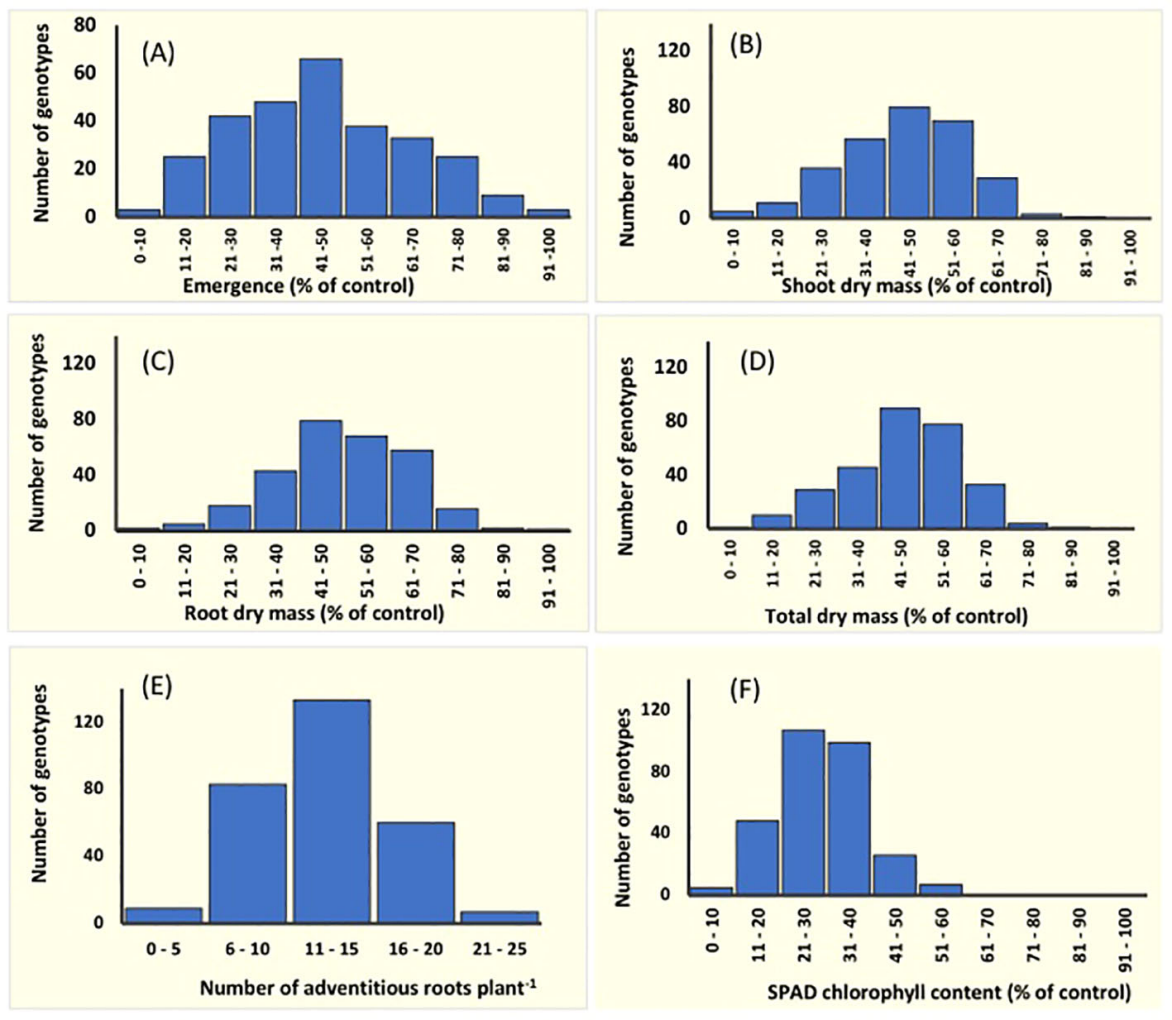
Frequency distribution for the variation in six traits in 292 mungbean mini−core collection genotypes. Data represent the per cent reduction compared with its control. Unadjusted phenotype data were used for adventitious root formation as there were no adventitious roots in their drained-controlled pots. At the germination stage: **(A)** seedling emergence, and at the seedling stage: **(B)** shoot dry mass, **(C)** root dry mass, **(D)** total dry mass, **(E)** adventitious root number, and **(F)** SPAD chlorophyll content on the first trifoliate leaves. Data for seedling emergence were recorded for seeds exposed to 4 days of soil waterlogging followed by 7 days of recovery. Shoot, root and total dry mass, and adventitious root number were recorded for seedlings (15 DAS) exposed to waterlogging for 8 days followed by 7 days of recovery. SPAD chlorophyll content was measured at the end of waterlogging treatment (23 DAS).

**Figure 2:**
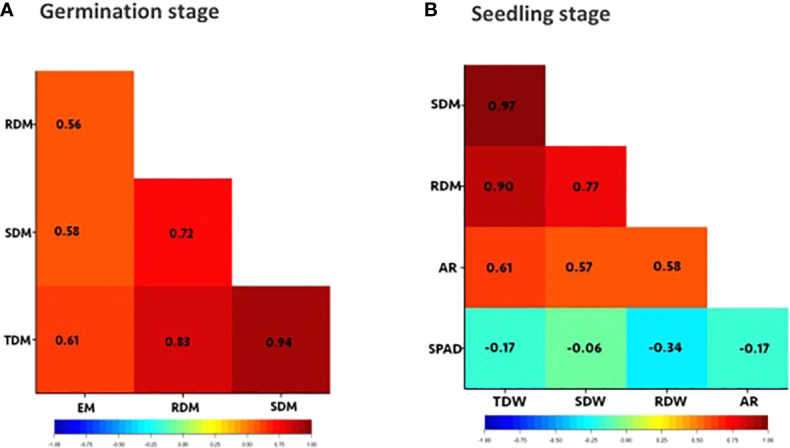
Correlation heatmaps for traits of interest. **(A)** germination stage: evaluated after 4 days of waterlogging and 7 days of recovery, **(B)** seedling stage: evaluated after 8 days of waterlogging from 15 days and 7 days of recovery. Evaluated traits: emergence (EM), shoot dry mass (SDM), root dry mass (RDM), total dry mass (TDM), number of adventitious roots (AR), SPAD chlorophyll content on the first trifoliate leaves (SPAD) at 23 DAS. Values within each cell are Pearson’s correlation coefficient. Significant correlations are indicated by orange and red cells. The blue and green cells indicate significant negative correlations.

Significant marker−trait associations were identified using three different criteria, including: (i) a GWAS threshold P−value equal to 1/m, where m represents the number of genotype markers analysed (Wang et al., 2012; Borrego-Benjumea et al., 2021); (ii) an FDR (false discovery rate) threshold; and (iii) a Bonferroni threshold, equal to 0.01/n, where n represents the number of genotype markers analysed. Manhattan plots illustrated the significance of markers associated with the measured traits (**Figures 1**, **2**). All annotated genes in the Vradiata_ver6 reference genome assembly (Kang et al., 2014) that were within average genome−wide LD decay distance of significantly associated SNP markers were identified as candidate genes for the corresponding putative transient waterlogging tolerance loci. Information on candidate gene ontology was sourced from Ensembl (plants.ensembl.org), AmiGO gene Ontology (amigo.geneontology.org) and UniProtKB (www.uniprot.org).

## Results

### Effect of transient waterlogging on soil redox potential

Soil redox potential was greatly reduced in the waterlogged treatments, indicating that oxygen had been depleted from the soil. Such hypoxic/anoxic conditions are experienced in the field as a result of waterlogging and affect crop growth by preventing aerobic cellular respiration in root tissues, which is required to generate sufficient energy for root growth and functioning (i.e. absorption and transport of water and nutrients to the shoot). At the germination stage, drained pots had a stable soil redox potential (400 ± 32 mV) throughout the experimental period. In contrast, in the waterlogged pots, the soil redox potential declined gradually to 208 ± 10 mV after 4 days of waterlogging, where it remained for 3 days before gradually increasing during the recovery period, reaching the drained control value at 10 DAS (**Supplementary Figure 1A**).

At the seedling stage, the drained pots had a stable soil redox potential (440 ± 19 mV) from the first day of waterlogging (15 DAS) to harvest (30 DAS). In contrast, the soil redox potential of the waterlogged pots gradually decreased, reaching its lowest point 220 ± 15 mV after 3 days of waterlogging (18 DAS) before gradually increasing to 250 ± 35 mV by the end of the waterlogging treatment (23 DAS) and reaching the control level after 6 days of recovery (**Supplementary Figure 1B**).

### Germination stage screening

#### Check genotypes

Check genotypes in the two cohorts [Cohort I (C−I) and Cohort II (C−II)] were analysed to understand the impact of phenotyping the mini−core collection in two separate cohorts for practicality reasons. Significant effects were detected for check genotypes (Gen), the transient waterlogging treatment (Treat) and their interaction (Treat × Gen and Treat × Gen × Cohort) for all traits, including emergence (%) and shoot, root, and total dry mass (all *P* ≤ 0.01; **Supplementary Table 5**). As the genotypic response to transient waterlogging was significantly greater than the cohort effect for the Treat x Gen x Cohort interactions (all *P* ≤ 0.01; **Supplementary Table 5**), data from the two cohorts were combined for analysis of all traits. Among the check genotypes, VI 4069 had the highest emergence (62%), followed by Celera II−AU (46%), VI 2537 (46%), VI 4954 (45%), and VI 2173 (38%), while BARIM−3, BARIM−6 and Jade−AU were the most susceptible (~20%) to waterlogging (**Supplementary Figure 2**).

#### Mini-core collection genotypes

Emergence and dry mass accumulation traits were significantly affected by Gen, Treat, and Treat × Gen interaction effects (*P* < 0.001 for all traits; **Table 1**). Transient waterlogging treatment had the greatest impact amongst the variables and reduced emergence by an average of 52% relative to the drained control. However, genetic effects were also prominent and a wide range of genetic variation was observed in the mini-core collection (**Supplementary Table 6**; **Figure 1A**), particularly for emergence (ranging from 0 – 90% under stress conditions) and root and total dry mass (ranging from 0 – 0.4 g and 0 – 1.1 g under stress conditions, respectively). As expected, seedling emergence under transient waterlogging was moderately correlated with the shoot dry mass (0.58), root dry mass (0.56) and total dry mass (0.61) (**Figure 2A**). All traits exhibited high broad-sense heritability: 81% for emergence, 83% for shoot dry mass, and 71% for root dry mass (**Supplementary Table 6**). Similarly, principal component loadings of seedling emergence under transient waterlogging correlated highly with shoot, root, and total dry mass (**Supplementary Table 7**). The three principal components had Eigenvalues of 3.14 (PC1), 1.05 (PC2) and 0.57 (PC3), accounting for 63% (PC1), 21% (PC2) and 11% (PC3) of total variability at germination.

**Table 1:** Analysis of restricted maximum likelihood (REML) of the mungbean mini-core population screened at the germination stage for emergence (%) and shoot, root, and total dry mass.

Variable	Source of variation	Genotype	Treatment	Treatment × Genotype
	n.d.f*	291	1	291
	d.d.f**	1384	1384	1384
Emergence (%)	Wald statistic	2760.63	9671.48	1803.99
	F statistic	9.49	9671.48	6.2
	F pr	<0.001	<0.001	<0.001
Total dry mass (g)	Wald statistic	3710.12	8570.59	2463.24
	F statistic	12.75	8570.59	8.46
	F pr	<0.001	<0.001	<0.001
Shoot dry mass (g)	Wald statistic	2757.35	9565.41	1826.80
	F statistic	9.48	9565.41	6.28
	F pr	<0.001	<0.001	<0.001
Root dry mass (g)	Wald statistic	1659.37	3497.50	1330.06
	F statistic	5.7	3497.5	4.57
	F pr	<0.001	<0.001	<0.001

*numerator degrees of freedom.

**denominator degrees of freedom.

The screening was undertaken in two cohorts, with the data combined and analysed to explore genetic variation in mungbean.

This study revealed that the tolerance of germplasm to transient waterlogging stress, as reflected by all traits except for root dry mass, significantly varied amongst contrasting geographic regions of origin (all *P* ≤ 0.01; **Supplementary Table 8**). However, this analysis was highly unbalanced, with 64% of genotypes from South Asia, 20% from Southwest Asia and 6.8% from Southeast Asia (**Supplementary Table 1**). Nonetheless, genotypes from South Asia and Oceanic Pacific had the highest emergence (51%, 50%) compared with those from Europe (29%) and Africa (35%).

### Seedling stage screening

#### Check genotypes

As with the previous germination growth stage, check genotypes were analysed to gauge homogeneity between the three phenotyping cohorts [cohort I (C−I), cohort II (C−II) and cohort III (C−III)]. Significant differences occurred between Gen, Treat, and Cohort variables, plus the Treat × Gen and Treat × Cohort interactions for all traits, but not for the Gen × Cohort or Treat × Gen × Cohort interactions (**Supplementary Table 9**). As the genotypic response to waterlogging was considerably greater than the cohort effect in the Gen × Cohort interaction, data from the three cohorts were once again combined for further analysis.

Transient waterlogging at the seedling stage caused notable reductions in shoot and root growth (*P* < 0.001) amongst the check genotypes. The extent of this biomass reduction varied substantially between genotypes (**Supplementary Figures 3A, B**). For example, smaller (yet statically significant, *P<* 0.001) reductions in root and shoot dry masses were observed in VI 2173, VI 2537, Jade−AU, and VI 4954, in contrast to genotypes such as Celera II−AU and BARIM−3, which experienced reduction up to three−fold for these traits. In terms of adventitious root development under transient waterlogging stress, Jade−AU and VI 4954 produced the largest number of new adventitious roots (20 roots on average), including surface roots, while BARIM−3 produced the least (8 roots on average) (**Supplementary Figure 3C**).

#### Mini−core collection genotypes

Effects of Gen, Treat, and the interaction of these two variables (i.e. Gen × Treat) significantly differed for all traits examined during the seedling growth stage, including dry mass (shoot, root, and total), adventitious root number, and SPAD chlorophyll content (all *P* < 0.001). Interestingly, Gen effects were stronger than the Treat or Treat × Gen effects for shoot, root, and total dry mass traits, whereas Treat had greater impact on adventitious root formation and SPAD chlorophyll content (refer to Wald statistics in **Table 2**).

**Table 2:** Analysis of restricted maximum likelihood (REML) of the mini-core collection genotypes screened in three cohorts at the seedling stage for shoot, root and total dry mass, adventitious root number and SPAD chlorophyll content.

Variable	Source of variation	Genotype	Treatment	Treatment × Genotype
Total dry mass (g)	n.d.f*	291	1	291
	d.d.f**	1258	1258	1258
	Wald statistic	1409.1	1302.8	296.3
	F statistic	4.8	1302.8	1.0
	F pr	<0.001	<0.001	<0.001
Shoot dry mass (g)	Wald statistic	1211.26	1032.57	270.72
	F statistic	4.1	1032.6	0.9
	F pr	<0.001	<0.001	<0.001
Root dry mass (g)	Wald statistic	1651.04	1320.04	354.3
	F statistic	5.7	1320.0	1.2
	F pr	<0.001	<0.001	<0.001
Adventitious root number	Wald statistic	662.06	6513.08	664.34
	F statistic	2.3	6513.1	2.3
	F pr	<0.001	<0.001	<0.001
SPAD chlorophyll content	Wald statistic	942.58	4532.3	538.6
	F statistic	3.2	4532.3	538.6
	F pr	<0.001	<0.001	<0.001

*numerator degrees of freedom.

**denominator degrees of freedom.

The cohort data were combined and analysed to explore the genetic variation.

A wide range of phenotypic responses were observed in response to transient waterlogging during this growth stage, indicating substantial variation in genetic stress tolerance within the mini−core collection. On average, transient soil waterlogging decreased shoot, root, and total dry mass by 50% after 8 days of stress compared to the drained controls (**Supplementary Table 6**). However, the overall ranges for dry mass were quite extensive. For example, reductions in root dry mass as small as 7−8% were observed in 2 genotypes (AGG325698 and AGG325738) while 22 genotypes reported reductions as large as 70−93% (**Figure 1**). Similarly, a wide range of phenotypic variation existed for SPAD chlorophyll content in the first trifoliate leaves by the end of the recovery period (i.e. 23 DAS; **Supplementary Table 6**; **Figure 1F**), although the average reduction was quite substantial (70%) relative to the drained controls (**Supplementary Table 6**). All four traits (dry masses and SPAD chlorophyll content) were highly heritable with values ranging from 70% to 81% (**Supplementary Table 6**). Strong positive correlations were present as expected between the dry mass traits under transient waterlogging stress (ranging from 0.77 – 0.97), but SPAD chlorophyll content was very weakly negatively correlated with all other traits (**Figure 2B**).

The majority (140 out of 292) of mini-core collection rapidly produced adventitious roots in the hypocotyl region near the soil surface. No adventitious rooting was observed in any of the drained control pots. The number of adventitious roots produced was somewhat consistent amongst those genotypes producing these root structures, with 133 genotypes producing 11–15 adventitious roots, which was similar to the overall average of 12 adventitious roots per genotype (**Supplementary Table 6**; **Figure 1E**). However, seven genotypes produced >20 adventitious roots (AGG325560, AGG325593, AGG325634, AGG325733, AGG32514, AGG325718, AGG325726). Overall, the formation of these root structures appeared to be moderately correlated with shoot dry mass (0.57), root dry mass (0.58) and total dry mass (0.61) (**Figure 2B**).

Additionally, the trait of adventitious root formation appeared to have moderate heritability of 56% (**Supplementary Table 6**), although this was the lowest reported for all traits at either growth stage. Some adventitious roots grew along the surface of the soil and visual observation identified nodules on some of these, but they were not measured.

In the PCA analysis, the first three components accounted for 87% of the total variation in the data (39% PC1, 30% PC2 and 17% PC3) (**Supplementary Table 7**). The individual contributions to total variance were highest for adventitious root numbers in PC1 and PC2 (0.62 and 0.76). However, for PC1, SPAD chlorophyll content had a negative contribution (–0.68), while it had a positive contribution in PC3 (0.57).

### Genetic diversity, population structure and LD analysis

**Figure 3:**
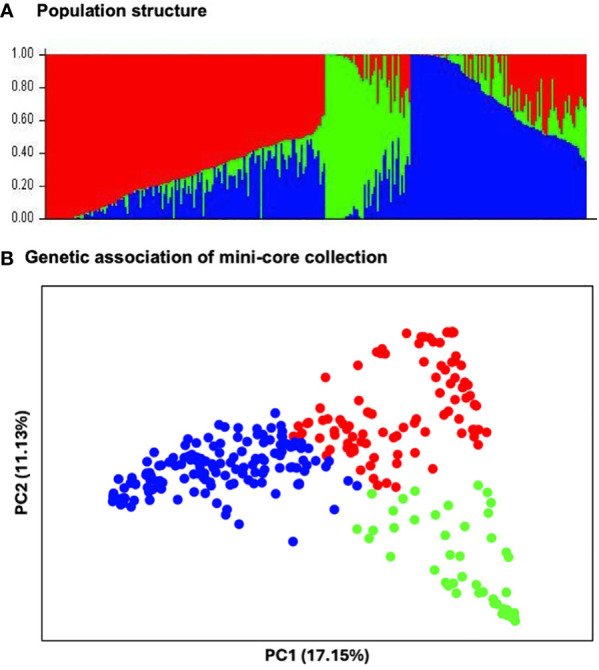
Population structure of 292 mungbean mini-core genotypes: **(A)** Classification of three populations using structure 2.3.4. The optimal number of clusters (k=3) is estimated based on the absolute value of the second-order rate of change in the likelihood distribution. Each vertical bar represents a single accession, and the length of each bar represents the proportion contributed by each population. The colour code indicates the distribution of mini-core genotypes to different populations: subpopulation 1 (red), subpopulation 2 (green) and subpopulation 3 (blue). **(B)** Genetic association of three subpopulations of mini-core collection genotypes based on 3,522 markers developed from DArT sequencing, revealed by a principal component analysis (PCA).

**Figure 4:**
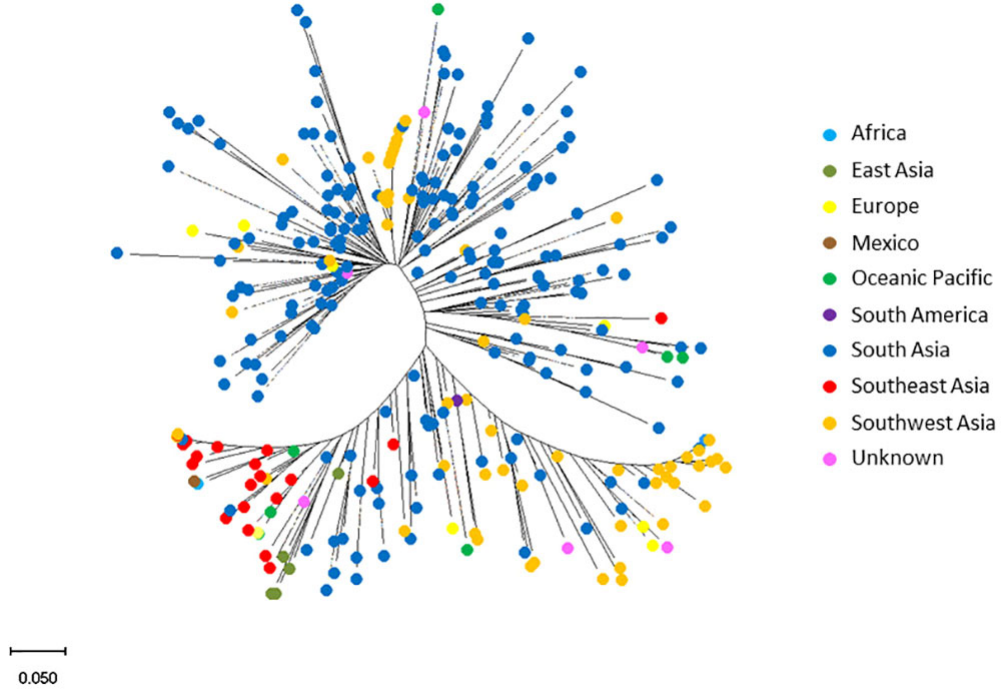
Diversity of mungbean mini-core collection genotypes. Neighbour-joining tree analysis of 292 mungbean mini core genotypes: 186 from South Asia, 57 from Southwest Asia, 20 from Southeast Asia, seven from Ocean Pacific, five from East Asia, two from Europe, two from Africa, two from South America and 11 of unknown origin. The analysis was based on a bootstrap consensus phylogenetic tree inferred from 100 replicates to represent the evolutionary history of the taxa analysed.

The STRUCTURE analysis revealed the presence of three distinct subpopulations (**Figure 3A**) comprising 97 genotypes, 46 genotypes and 149 genotypes, respectively. These subpopulations neatly resolved into distinct groups in the PCA, in which the first two PCs explained 28.36% of the total observed variation (**Figure 3B**). Furthermore, the phylogenetic tree also identified three major clusters. The three clusters did not correspond to the geographic regions of origin: South Asian genotypes were found across the entire population but predominantly in subpopulation 1. Genotypes from Africa, East Asia, Europe, Mexico, Oceanic Pacific, Southeast Asia, and Southwest Asia were in subpopulation 3 (**Figure 4**).

**Figure 5:**
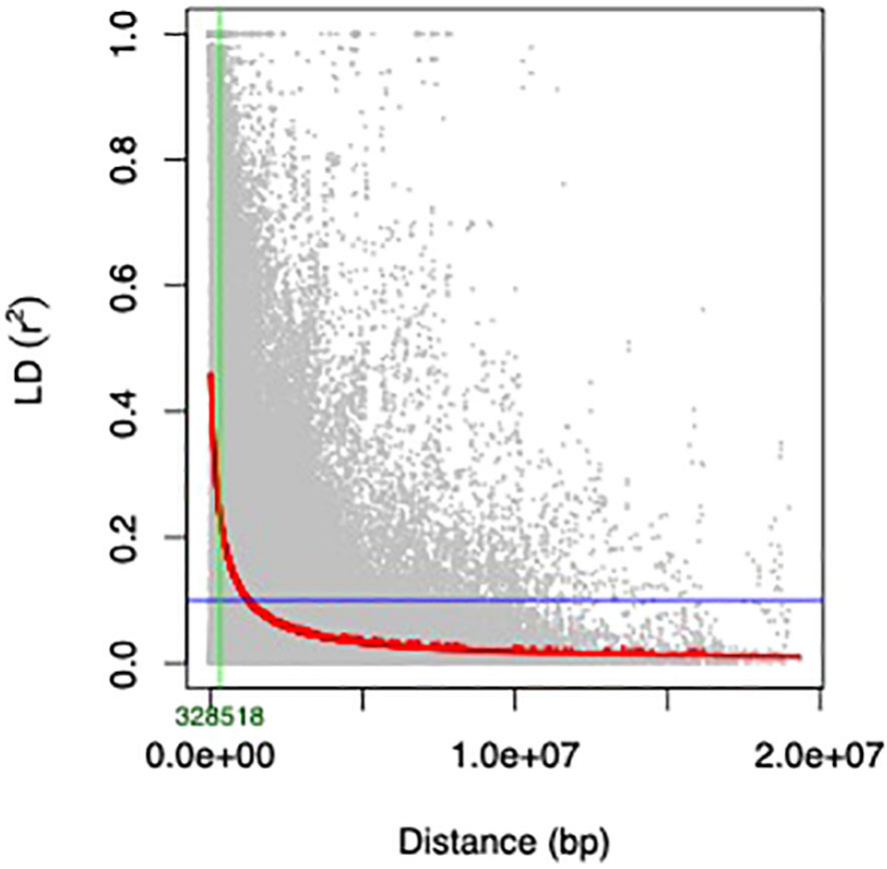
The LD decay was estimated from single-nucleotide polymorphism (SNP) genotypes of 292 mungbean mini-core collection genotypes. The curve represents the average LD of 11 chromosomes of the mini core population. The LD decay at r^2^ = 0.1 with ~328 Kb.

Using a threshold r^2^ value of 0.1, genome−wide LD was found to decay at 328,518 bp (**Figure 5**). This distance exceeded the average distance between SNPs on all chromosomes (**Supplementary Table 4**), indicating that the 3,522 filtered SNPs (MAF ≤ 0.05) were adequate for GWAS in the current study.

### GWAS and candidate gene discovery

**Figure 6:**
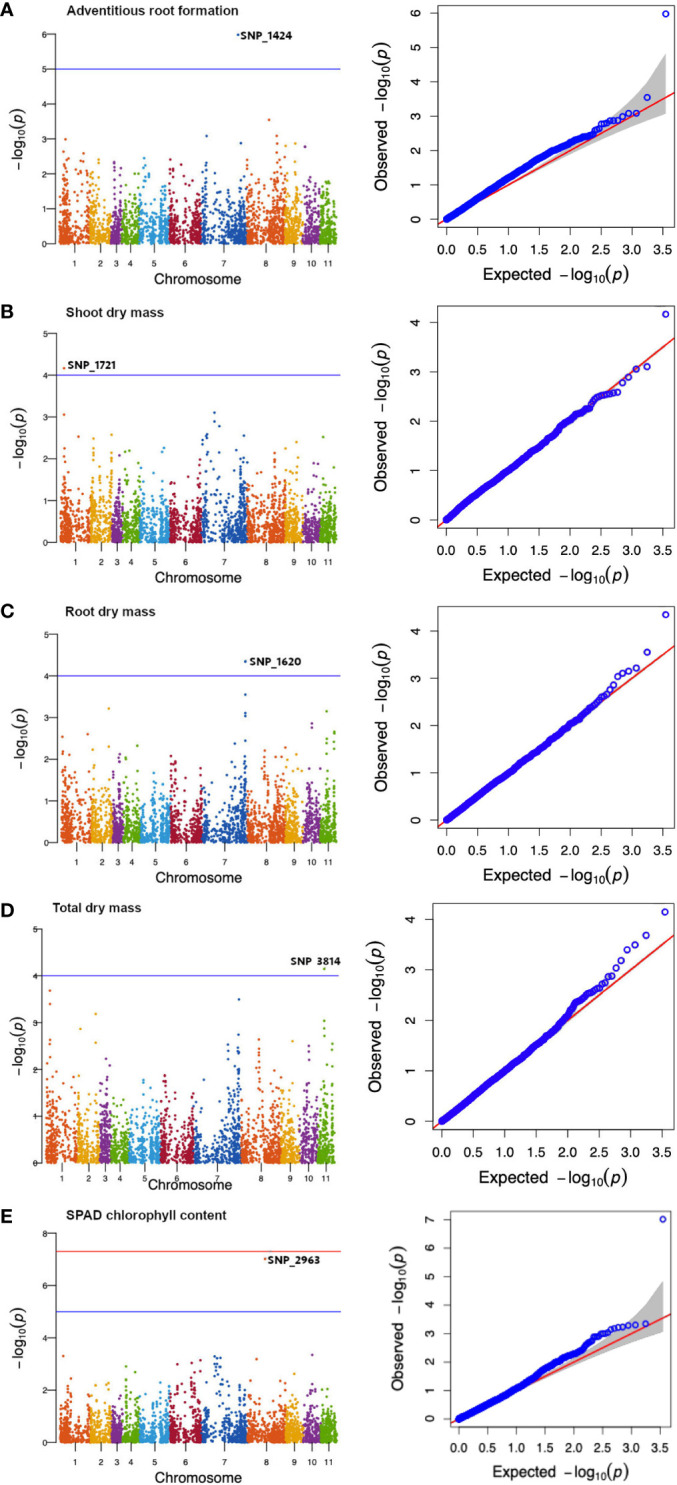
Manhattan diagrams of genome−wide association mapping results for mungbean mini-core collection at the seedling stage: **(A)** adventitious root formation, **(B)** shoot dry mass, **(C)** root dry mass, **(D)** total dry mass and **(E)** SPAD chlorophyll content. The x-axis indicates the SNP location along the 11 mungbean chromosomes, while the y-axis represents –log_10_(p) for the p-value of the marker-trait association. The red horizontal line represents the genome-wide significant SNPs threshold of p-values = 5 × 10^-8^. The blue horizontal line denotes the 5% Bonferroni-corrected threshold for 3,522 markers.

A total of five SNPs on chromosomes 1, 7, 8, and 11 (**Table 3**) were associated with transient waterlogging tolerance at the seedling stage. Adventitious root formation was associated with a SNP (SNP_1424) located on chromosome 7 within an exon of an FGGY carbohydrate kinase domain-containing protein gene (**Figure 6A**; **Table 3**). Furthermore, several plausible candidate genes were identified within the LD decay distance of significant SNP associations for the traits of adventitious root formation (**Supplementary Table 10**).

**Table 3:** Significantly associated SNPs and corresponding candidate genes for waterlogging-tolerant traits identified through GWAS.

SNP ID/Trait	Gene ID	Chr	Alleles	Position (bp)	P-value	LOD score	R^2^ value	FDR adjusted P-values	MAF	Call Rate	Protein name of candidate gene
Adventitious roots number											
SNP_1424	LOC106768660	7	G/A	44922869	1.04e-06	5.97	0.0227	0.00369	0.18	0.97	FGGY carbohydrate kinase domain-containing protein
Shoot dry mass											
SNP_1721	LOC111241851	1	T/C	4402474	‘6.80e-05	6.8	0.0489	0.239	0.37	1.0	mRNA-uncharacterized LOC111241851
Root dry mass											
SNP_1620	LOC106767922	7	G/A	52673662	4.51e-05	4.51	0.0244	0.159	0.18	0.96	probable caffeoyl-CoA O-methyltransferase At4g26220
Total dry mass											
SNP_3814	LOC106777236	11	A/G	7491697	7.11e-05	7.11	0.0612	0.250	0.11	1.0	MORC family CW-type zinc finger protein 3 and zinc finger protein 2B
SPAD chlorophyll content											
SNP_2963	LOC106770822	8	C/T	20831249	9.64e-08	7.01	0.0591	0.0033	0.24	1.0	3-oxoacyl-[acyl-carrier-protein] synthase, mitochondrial

Chrom represents Chromosome, FDR represents False Discovery Rate and MAF represents Minor Allelic Frequency.

The shoot dry mass trait was associated with a genomic region on chromosome 1 (SNP_1721) containing mRNA-uncharacterised protein LOC111241851 (**Figure 6B**; **Table 3**). The root dry mass trait was associated with the coding sequence of Caffeoyl-CoA O-methyltransferase At4g26220, also referred to as AtCCoAOMT (**Figure 6C**; **Table 3**). Meanwhile the total dry mass of mungbean plants under waterlogged condition was associated with the MORC family CW-type zinc finger protein 3 and zinc finger protein 2B (**Figure 6D**; **Table 3**). Finally, the SPAD chlorophyll content on the first trifoliate leaves was associated with a SNP (SNP_2963) located on chromosome 8 (**Figure 6E**; **Table 3**) within an exon of an 3−oxoacyl−[acyl-carrier-protein] synthase, mitochondrial, gene. FAR1−RELATED SEQUENCE 5−like and alcohol dehydrogenase class−3 were among other candidate genes observed within LD decay distance of the SNP (**Supplementary Table 11**). The current study did not identify the significant association between DArT-seq markers and the phenotypic traits of emergence.

## Discussion

This study represents the first report of genomic variation for transient soil waterlogging tolerance in a diverse collection of mungbean germplasm and contributes to a broader understanding of transient waterlogging tolerance in pulses. The pot−based screening methodology (Kyu et al., 2021) was adopted to create uniform hypoxic conditions, identifying variation in waterlogging tolerance within the mungbean mini−core germplasm collection at the germination and seedling growth stages. All traits of interest related to waterlogging stress tolerance and recovery (including emergence; adventitious root formation; shoot, root, and total dry mass; and SPAD chlorophyll content of leaves) exhibited a wide range of phenotypes. The high broad-sense heritability estimates for each of these traits indicates that this variation is largely due to genetic effects and demonstrates the possibility of selecting for waterlogging tolerance during breeding of climate−resilient mungbean cultivars. Furthermore, we identified multiple genotypes within the mini−core collection that may be used as parental donors for transient waterlogging stress tolerance loci in breeding due to their high emergence (AGG325612, AGG325667, AGG325523), production of root dry mass (AGG325758, AGG325732, AGG325695, AGG325734, AGG325761), and development of large numbers of adaptative adventitious roots (AGG325560, AGG325593, AGG325733, AGG325634, AGG325718, AGG325514, AGG325726) during transient waterlogging. While the majority of these valuable genotypes are of South Asian origin, the discovery of two genotypes from African and Oceanic Pacific origins demonstrates the potential of discovering adaptive genetics (including potentially unique loci) from multiple regions and reinforces the value of broad germplasm screening in pre−breeding research.

Understanding the strategies used by a species to cope with transient waterlogging stress and identifying genetic variation associated with transient waterlogging tolerance traits are prerequisites to breed for tolerance. Considerable variation in waterlogging tolerance exists within and between grain legume species. For example, faba bean (*Vicia faba* L.) produces adventitious roots and aerenchyma (increasing root porosity by 9%); thus, it is more tolerant to short-term waterlogging than yellow lupin (*Lupinus luteus* L.), grass pea, narrow-leaf lupin (*Lupinus angustifolius* L.), chickpea (*Cicer arietinum* L.) and lentil (*Lens culinaris* Med. subsp. *culinaris*) (Solaiman et al., 2007). Limited research has explored the mechanisms for tolerance in mungbean to date, and studies that have been conducted have largely explored coping strategies during reproductive development (Van Haeften et al., 2023). Here, we have examined how the species responds to stress during earlier stages of growth.

We confirmed that mungbean seeds do not germinate during transient waterlogging, and that variation in transient waterlogging tolerance was related to maintaining seed viability under hypoxia and subsequent emergence on the release of hypoxia. Tolerant genotypes (i.e. AGG325523, AGG325612, AGG325667) had similar emergence percentages to the drained controls after removing the stress, but sensitive genotypes (i.e. AGG325528, AGG325569, AGG325491) failed to emerge and died (**Supplementary Table 12**), similar to barley germplasm screened for waterlogging tolerance (Takeda and Fukuyama, 1987). In other legumes, such as soybean, waterlogging tolerance is related to the thickness of the seed aleurone layer, with tolerant genotypes absorbing water more slowly than sensitive genotypes (Tian et al., 2005; Sato et al., 2019). Moreover, Powell and Matthews (1979) reported that pea seeds rapidly absorbed water under soil waterlogging, but the inhibited respiration rate decreased the formation of adenosine triphosphate (ATP) (Johnson et al., 1989), resulting in poor seed viability and germination.

**Figure 7:**
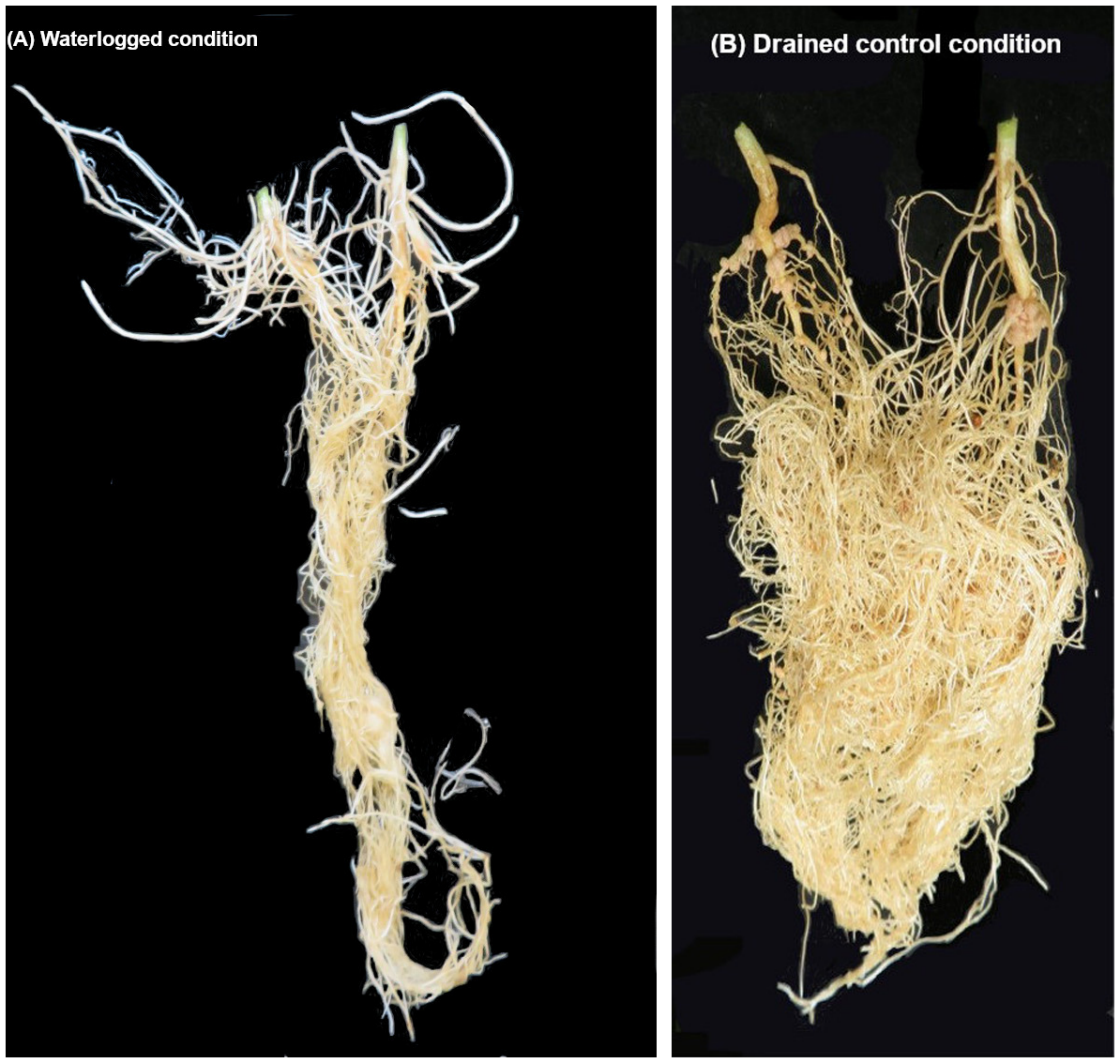
Mungbean (cv. Jade−AU) root development under waterlogged and drained control conditions: **(A)** formation of adventitious roots in mungbean seedlings in response to 8 days of transient soil waterlogging, and **(B)** root development under drained control conditions. Note the presence of new white adventitious roots on the waterlogged plants.

At the seedling stage, transient waterlogging stress reduced shoot and root relative growth (% of control) and SPAD chlorophyll content in mungbean. In response to the stress, tolerant germplasm produced adventitious roots primarily in the hypocotyl region (**Figure 7**), as rapidly as 2–4 days after the onset of transient waterlogging, including some of which grew along the surface of the soil. As a result, genotypes that formed adventitious roots in response to transient waterlogging had higher root and shoot dry mass and SPAD chlorophyll content than genotypes that did not form such new roots (**Supplementary Table 13**). Adventitious root formation is a significant trait in many waterlogging− and flood−tolerant plant species (Jackson and Armstrong, 1999), including soybean (Valliyodan et al., 2014; Kim et al., 2015), common bean (Soltani et al., 2018), cotton (Zhang et al., 2021), barley (Borrego-Benjumea et al., 2021) and wild maize (teosinte) (Mano et al., 2005). In contrast to the primary root system that has limited capacity for gas exchange at depth under waterlogged conditions, newly formed adventitious roots avoid hypoxia/anoxia due to their location above the soil, at the soil surface, and/or at shallow soil depths, and thereby enhance the overall uptake of oxygen into the root system (Gonin et al., 2019). Additionally, they contain aerenchyma (i.e. spongy tissues that act as internal channels for oxygen transport) and may also have specialised structures called radial oxygen loss (ROL) barriers that minimize the loss of oxygen to the rhizosphere and efficiently direct it across and along the roots to the meristematic tissues (Yamauchi et al., 2018). Consequently, adventitious roots are able to compensate, at least to some extent, for the impaired function of the primary root system and enable plants to continue acquiring water and nutrients (Colmer and Greenway, 2011; Voesenek and Bailey-Serres, 2013; Steffens and Rasmussen, 2016), which contributes to plant survival under stress. In addition to differences in adventitious roots, a range of phenotypic variation was observed more generally for root systems; some genotypes had short taproots and a limited number of lateral roots (i.e. AGG325470, AGG325474, AGG325479).

Several studies have recently identified genetic loci and candidate genes associated with waterlogging tolerance in model pulses. In common bean, GWAS analysis using ~150 K SNPs identified candidate genomic regions at Pv08/1.6 Mb and Pv02/41 Mb relating to physiological responses of germination rate and root weight under waterlogging (Soltani et al., 2017). These two regions were also identified in soybean QTLs for waterlogging tolerance, indicating that they might control the evolutionary pathway for stress tolerance in pulses. Ye et al. (2018) reported that dominant alleles at *qWT_Gm03* controlled waterlogging tolerance at the vegetative stage in soybean. However, beyond these findings, it is unclear which genetic pathways contribute to waterlogging tolerance in pulses (Soltani et al., 2017).

The inheritance of waterlogging tolerance in mungbean was dissected in this study by marker−trait associations for the first time. This was made possible by the recent development of new genetic and genomic resources for mungbean, including a reference genome assembly (Kang et al., 2014) and DArTseq markers for a mini−core germplasm collection (Schafleitner et al., 2015). One of the main advantages of GWAS is that it can assess greater genetic/allelic diversity for a species using a natural population than is possible using bi−parental populations for linkage mapping (Korte and Farlow, 2013). In addition, historical recombination events within natural populations can provide greater mapping resolution than achieved using genetic populations in linkage mapping, provided that low levels of linkage disequilibrium (i.e., small LD decay distances) exist in the GWAS mapping population and a large number of genetic markers (saturating the entire genome) are used (Korte and Farlow, 2013).

At the germination stage, we unfortunately did not identify any genomic regions associated with emergence after four days of transient waterlogging, despite this trait having strong heritability. The reason for this is very likely to be the small number of SNPs used in our analysis due to stringent quality filtering. Given the strong heritability we observed (H^2 = ^0.81), we suspect it very likely that strong associations would be found in the mini-core collection of mungbean germplasm in future studies that are able to utilise higher densities of markers, for example, derived through whole genome resequencing.

At the seedling stage, a number of candidate genes were identified within five associated genomic regions. Although more genetic associations are very likely to be found in future studies employing higher densities of molecular markers, the identified genomic regions and associated markers identified in this study will be valuable as an initial foundation for isolating transient waterlogging tolerance genes to improve mungbean breeding. Additionally, the corresponding candidate genes may enhance our understanding of potential strategies and genetic pathways that provide waterlogging tolerance in mungbean (**Supplementary Tables 10, 11**).

The GWAS analysis revealed that adventitious root formation under waterlogging stress was possibly associated with FGGY carbohydrate kinase domain−containing. Interestingly, this suggests a link between carbohydrate metabolism and adventitious root development during transient waterlogging, in addition to the shift in metabolism from aerobic to anaerobic pathways and the availability of soluble sugars during low oxygen stress (Bailey-Serres and Voesenek, 2010). In sorghum seedlings, the FGGY carbohydrate kinase family shares the same phylogenetic nodes with plant−growth−promoting rhizobacteria (PGPR), including several phytobeneficial and desired traits such as increased production during biotic or abiotic stress (Etesami and Maheshwari, 2018). Further investigations of the biological function(s) in plants of FGGY are needed (Singh et al., 2017) and, as highlighted here, should include the potential link to adventitious rooting during waterlogging stress in mungbean. Several other plausible candidate genes related to abiotic stress were also identified for this trait, however, and should similarly be explored further (**Supplementary Table 10**). For example, AAA−ATP *At5g57480* has novel ATPase activity that is associated with the formation of hypocotyl-derived adventitious roots in cucumber (Xu et al., 2018).

We identified that Caffeoyl-CoA O-methyltransferase At4g26220, also referred to as AtCCoAOMT, is associated with the root dry mass for the waterlogging tolerance in mungbean. GO annotations indicate that AtCCoAOMT enables O-methyltransferase activity (Burge et al., 2012) and S-adenosylmethionine-dependent methyltransferase activity (Tang et al., 2019). The AtCCoAOMT protein is an enzyme active in the phenylpropanoid pathway within plants, which is integral to the production of various secondary metabolites, including lignin, flavonoids, and phytoalexins, all of which serve vital functions in plant defense, structural integrity, and response to environmental stressors. Lignin, in particular, is essential for maintaining cell structure and enhancing resistance to both biotic and abiotic stresses (Bhuiyan et al., 2009; Weng et al., 2010; Srivastava et al., 2015). O-Methylation plays a key role in lignin biosynthesis, stress tolerance, and disease resistance in plants (Lam et al., 2007). The O-methyltransferase genes exhibit diverse responses to environmental stresses and developmental processes, including salt stress and fibre development in cotton (Hafeez et al., 2021), lodging resistance and feedstock quality associated with lignin content in wheat (Nguyen et al., 2016), and putative roles in development and stress tolerance such as low temperature, hormone, and drought stress in peanut (Cai et al., 2023).

Our GWAS analysis revealed that the total dry mass of mungbean plants under waterlogged condition is associated with the genes encoding MORC family CW-type zinc finger protein 3 and zinc finger protein 2B (**Table 3**). Recent studies reported that microrchidia (MORC) proteins are a family of evolutionarily conserved GHKL-type ATPases involved in chromatin compaction and gene silencing. Furthermore, the MORC-mediated repression of gene expression is particularly important under conditions of stress (Zhong et al., 2023). The ORC proteins also act downstream of DNA methylation to suppress gene expression and are also involved in plant immunity — protecting plants against potential pathogens by interacting with plant resistance proteins (Kang et al., 2008; Moissiard et al., 2012). Additionally, GO annotations indicate that MORC family CW-type zinc finger protein 3 and zinc finger protein 2B proteins are located in the nucleus and enable ATP hydrolysis (Tang et al., 2019) and zinc ion binding activities (Burge et al., 2012). Enabling of ATP hydrolysis and zinc ion binding activities in plants under waterlogged stress involved complex molecular mechanisms that help plants cope with adverse environmental conditions. ATP hydrolysis activity and zinc ion binding play crucial roles in plant adaptation to abiotic stress by providing energy for cellular processes, maintaining ion homeostasis, regulating enzyme activity, and enhancing antioxidant defense mechanisms (Li et al., 2022). Understanding the interplay between ATP hydrolysis and zinc ion binding could provide insights into the molecular mechanisms underlying plant responses to waterlogging stress and inform strategies for improving plant resilience and productivity in waterlogged conditions.

In the current study, an *OXSM* gene that encodes a 3−oxoacyl−[acyl−carrier−protein] synthase mitochondrial protein and beta-ketoacyl synthetase (**Table 3**) was identified as a possible candidate gene related to leaf chlorophyll content during transient waterlogging. There are three isoforms of ketoacyl−[acp] synthase, namely *KASI*, *KASII*, and *KASIII*. In Arabidopsis, a T−DNA insertion mutant, *KASI* showed multiple morphological defects, including chlorotic and curly leaves, reduced fertility, and semi−dwarfism, demonstrating pleiotropic effects of FA synthesis on plant growth (Wu and Xue, 2010). The current study observed chlorotic, curly leaves and semi−dwarf plants in the waterlogging treatment. However, further studies are needed to confirm what type of ketoacyl−[acp] synthase is involved in the tolerance of mungbean to transient waterlogging stress, especially in the chlorophyll content and photosynthesis pathway. Other proteins within the estimated LD length include regulating plant immunity (**Supplementary Table 11**).

In summary, this study has identified useful phenotypic variation for transient waterlogging-tolerance traits within the mungbean mini−core germplasm collection and has contributed to an improved understanding of the genetic control of waterlogging tolerance during germination and seedling development. Together, these findings provide new insights into the significance of transient waterlogging tolerance traits in mungbean, such as adventitious root formation, and genomic regions and candidate genes associated with transient waterlogging tolerance. Further studies are required to validate these results and to determine the correlation between the temperature−controlled glass house condition in this study and field conditions.

## Conclusion

The genetic diversity for transient waterlogging tolerance in the mungbean mini−core collection was explored under controlled conditions during germination and seedling growth stages using GWAS. Efficient screening methodology helped discriminate between susceptible and tolerant germplasm and identify key traits related to tolerance, such as the formation of adventitious roots in response to experiencing transient waterlogging. Thirty−seven genotypes were identified as being tolerant at both developmental stages, with 20 (7%) genotypes being tolerant at germination and 17 (6%) genotypes at the seedling stage. These genotypes could be used as donors for waterlogging tolerance in breeding programs and enable the development of new varieties with favourable combinations of alleles at both stages of plant development. Nevertheless, the genomic associations and corresponding candidate genes identified through GWAS in this study strengthen our understanding of the genetic mechanisms underlying transient waterlogging tolerance in mungbean. Furthermore, the significantly associated SNPs may be used to design robust molecular markers for future marker-assisted breeding of climate−resilient cultivars that can withstand waterlogging. This will be imperative for mitigating the detrimental effects of waterlogging stress on crops and reducing losses for mungbean growers.

## Data availability statement

The datasets presented in this study can be found in online repositories. The repositories can be found in the **Supplementary Material**.

## Author contributions

KK: Conceptualization, Data curation, Formal analysis, Investigation, Methodology, Software, Writing – original draft. CT: Software, Supervision, Writing – review & editing. CD: Conceptualization, Resources, Writing – review & editing. AM: Supervision, Writing – review & editing. TC: Conceptualization, Methodology, Project administration, Resources, Supervision, Validation, Visualization, Writing – review & editing. KS: Conceptualization, Methodology, Project administration, Resources, Supervision, Validation, Visualization, Writing – review & editing. WE: Conceptualization, Data curation, Formal analysis, Funding acquisition, Methodology, Project administration, Resources, Supervision, Validation, Visualization, Writing – review & editing.

## Supplementary material

Supplementary Figure 1 Soil redox potential during **(A)** germination stage, **(B)** seedling stage in the mini-core collection genotypes under waterlogged (WL) and drained (Control) conditions. At germination, WL was imposed immediately after sowing for 4 days, followed by 7 days of recovery, with the experiment terminating at 11 DAS. At the seedling stage, WL was imposed at 15 DAS, followed by 7 days of recovery, with the experiment terminating at 30 DAS. The arrows indicate the first day of waterlogging and the first day of recovery after the end of the WL treatment. The data are means of six soil redox potential for each treatment in each cohort. Vertical bars represent standard errors ( ± SE) of the mean.

Supplementary Figure 2 Effect of transient waterlogging (WL) at the germination stage on the emergence (%) of eight check genotypes from the mungbean mini-core collection screened in two cohorts [Cohort I (C-I) and Cohort II (C-II)]. The check genotypes were replicated five times in each cohort. WL was imposed immediately after sowing for 4 days, followed by 7 days of recovery, with the experiment terminating at 11 DAS. The data are means for each genotype in each treatment; vertical bars represent standard errors ( ± SE) and least significant differences (LSD) at *P* = 0.05 for genotype.

Supplementary Figure 3 Effect of transient waterlogging (WL) on **(A)** shoot dry mass, **(B)** root dry mass, and **(C)** adventitious root formation of eight check genotypes from the mungbean mini-core collection screened under transient waterlogging in three cohorts [C-I, C-II, C-III] at the seedling stage. WL was imposed at 15 DAS, followed by 7 days of recovery, with the experiment terminating at 30 DAS. The data are means for each genotype in each treatment; vertical bars represent standard errors ( ± SE) and LSD at *P* = 0.05 for genotype.
