# Supplementary figures and images for: Genetic diversity and candidate genes for transient waterlogging tolerance in mungbean at the germination and seedling stages
Source: Front Plant Sci. 2024 Mar 21;15:1297096. doi: 10.3389/fpls.2024.1297096 (PMC10996369; doi:10.3389/fpls.2024.1297096)

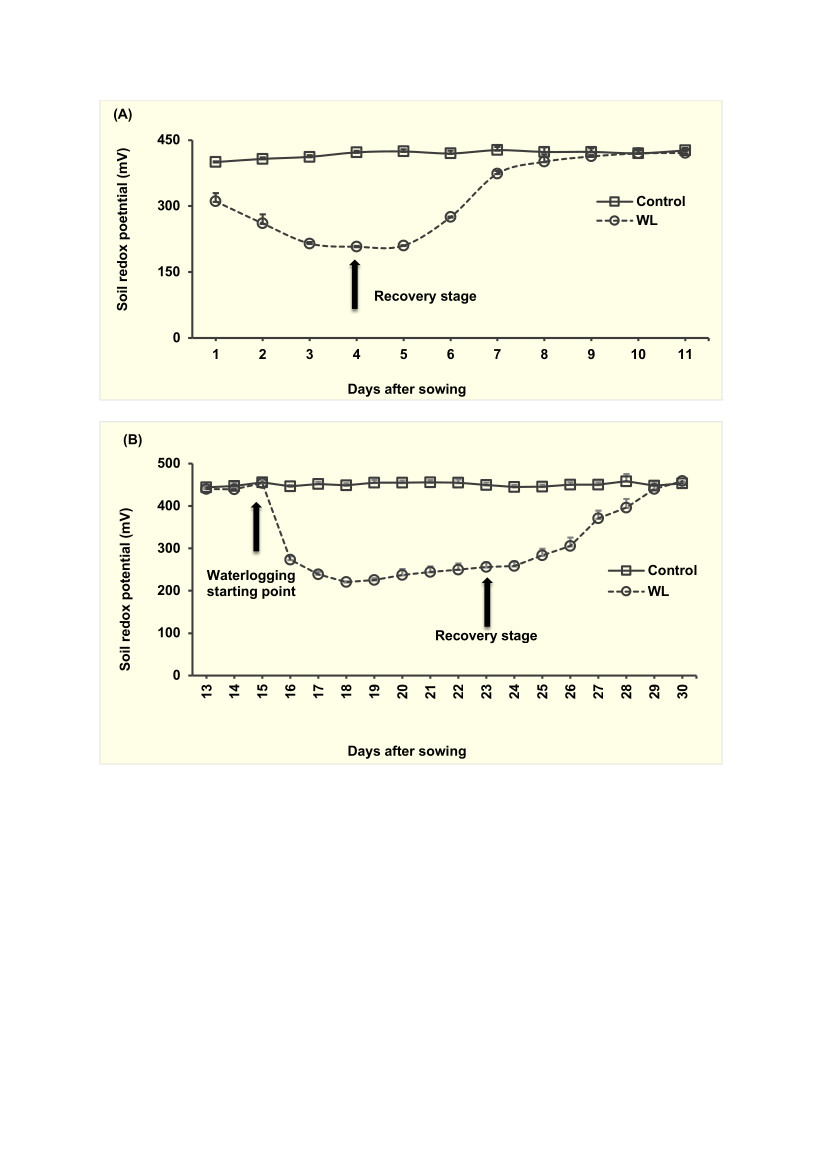

Supplement: Supplementary Figure 1 — Soil redox potential during (A) germination stage, (B) seedling stage in the mini-core collection genotypes under waterlogged (WL) and drained (Control) conditions. At germination, WL was imposed immediately after sowing for 4 days, followed by 7 days of recovery, with the experiment terminating at 11 DAS. At the seedling stage, WL was imposed at 15 DAS, followed by 7 days of recovery, with the experiment terminating at 30 DAS. The arrows indicate the first day of waterlogging and the first day of recovery after the end of the WL treatment. The data are means of six soil redox potential for each treatment in each cohort. Vertical bars represent standard errors ( ± SE) of the mean. [file Image_1.jpeg]

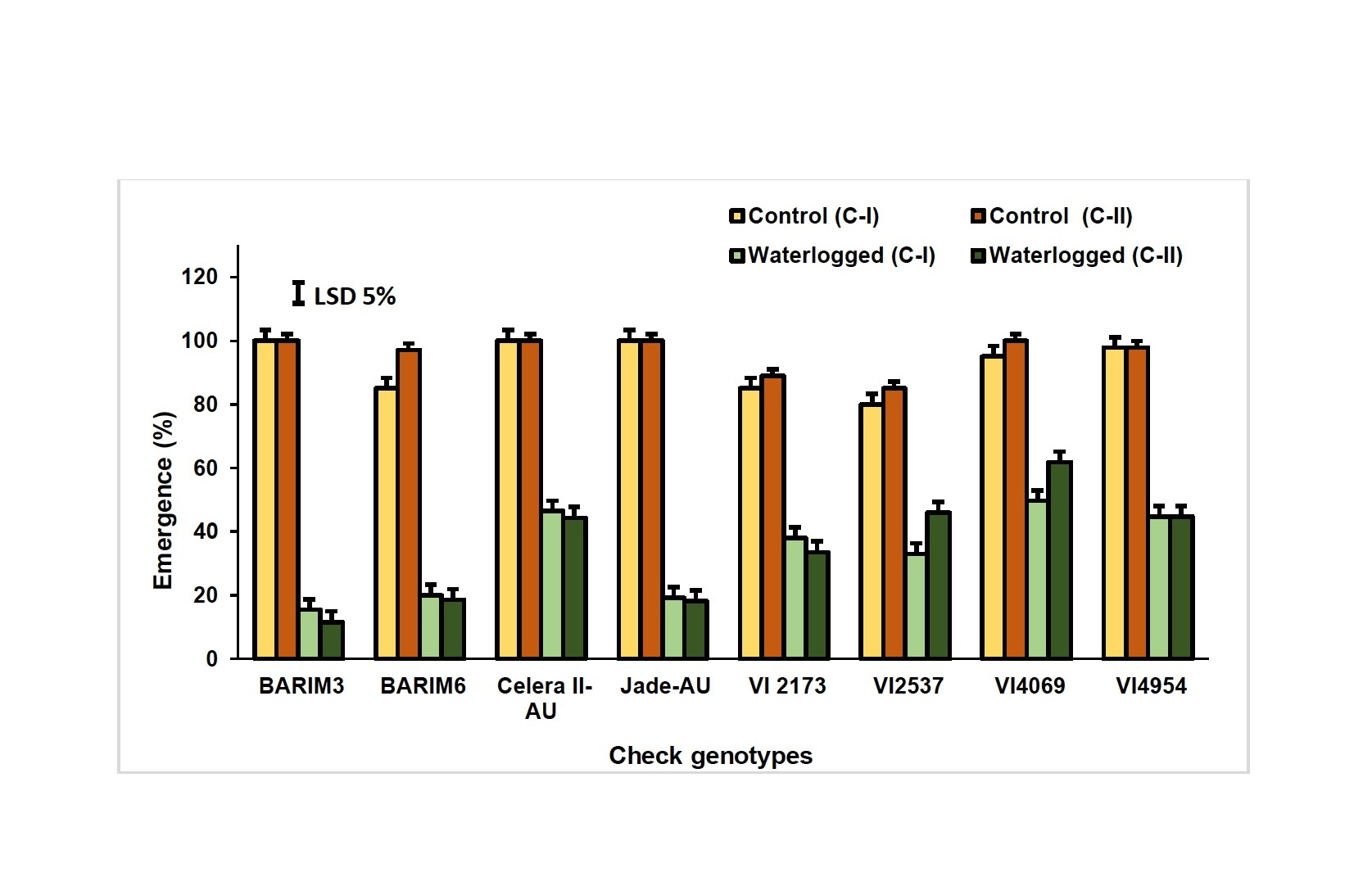

Supplement: Supplementary Figure 2 — Effect of transient waterlogging (WL) at the germination stage on the emergence (%) of eight check genotypes from the mungbean mini-core collection screened in two cohorts [Cohort I (C-I) and Cohort II (C-II)]. The check genotypes were replicated five times in each cohort. WL was imposed immediately after sowing for 4 days, followed by 7 days of recovery, with the experiment terminating at 11 DAS. The data are means for each genotype in each treatment; vertical bars represent standard errors ( ± SE) and least significant differences (LSD) at P = 0.05 for genotype. [file Image_2.jpeg]

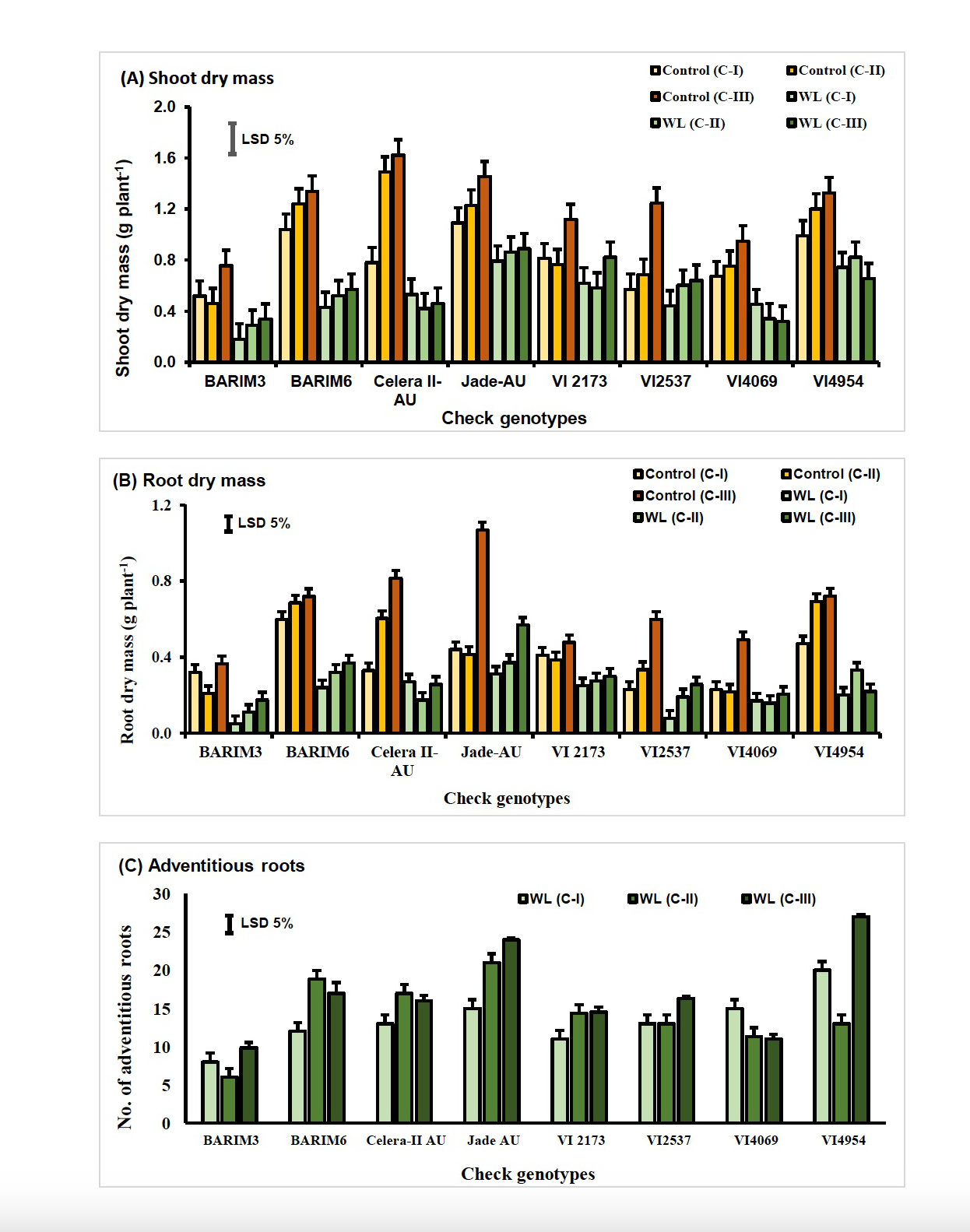

Supplement: Supplementary Figure 3 — Effect of transient waterlogging (WL) on (A) shoot dry mass, (B) root dry mass, and (C) adventitious root formation of eight check genotypes from the mungbean mini-core collection screened under transient waterlogging in three cohorts [C-I, C-II, C-III] at the seedling stage. WL was imposed at 15 DAS, followed by 7 days of recovery, with the experiment terminating at 30 DAS. The data are means for each genotype in each treatment; vertical bars represent standard errors ( ± SE) and LSD at P = 0.05 for genotype. [file Image_3.png]
