# Supplementary material for: Genetic diversity and candidate genes for transient waterlogging tolerance in mungbean at the germination and seedling stages
Source: Front Plant Sci. 2024 Mar 21;15:1297096. doi: 10.3389/fpls.2024.1297096 (PMC10996369; doi:10.3389/fpls.2024.1297096)
Supplement: Supplementary file 4 [file DataSheet_1.docx]

Supplementary Table 1| Distribution of regional origins of mungbean mini−core collection genotypes.

| **Variable** | **Region** |  | **No. genotypes** | **Percentage (%)** |
| --- | --- | --- | --- | --- |
| Region | Africa | (AFR) | 2 | 0.7 |
|  | Mexico | (MA) | 1 | 0.3 |
|  | East Asia | (EA) | 5 | 1.7 |
|  | Europe | (EUR) | 2 | 0.7 |
|  | Oceanic Pacific | (OP) | 7 | 2.4 |
|  | South Asia | (SA) | 186 | 63.7 |
|  | Southeast Asia | (SEA) | 20 | 6.8 |
|  | South America | (SM) | 1 | 0.3 |
|  | Southwest Asia | (SWA) | 57 | 19.5 |
|  | Not available | (NA) | 11 | 3.8 |

Supplementary Table 2| The analysis of soil samples. Before analysis, the soil samples were randomly collected and dried in the 60°C soil drying room for 5 days and sieved with 2 mm sieving machine. The data represent the means of four replications.

| **Name** | **Unit** |  |
| --- | --- | --- |
| Colour |  | BRGR |
| Gravel | % | 0 |
| Texture |  | 3 |
| Ammonium nitrogen | mg kg^–1^ | 1.7 |
| Nitrate nitrogen | mg kg^–1^ | 20 |
| Phosphorus Colwell | mg kg^–1^ | 15 |
| Potassium Colwell | mg kg^–1^ | 554 |
| Sulphur | mg kg^–1^ | 25 |
| Organic carbon | % | 0.3 |
| Conductivity | dS m^–1^ | 0.5 |
| pH (CaCl_2_) |  | 7.8 |
| pH (H_2_O) |  | 8.5 |
| DTPA copper | mg kg^–1^ | 2.1 |
| DTPA iron | mg kg^–1^ | 9.9 |
| DTPA manganese | mg kg^–1^ | 16.0 |
| DTPA zinc | mg kg^–1^ | 0.7 |
| Exchangeable aluminium | meq 100 g^–1^ | 0.1 |
| Exchangeable calcium | meq 100 g^–1^ | 8.6 |
| Exchangeable magnesium | meq 100 g^–1^ | 3.2 |
| Exchangeable potassium | meq 100 g^–1^ | 1.2 |
| Exchangeable sodium | meq 100 g^–1^ | 2.2 |
| Boron hot CaCl_2_ | mg kg^–1^ | 2.7 |

Supplementary Table 4| Coverage of SNPs per individual mungbean chromosome before and after filtering.

| **Chromosome** | **Before filtering** | | | **After filtering** | | |
| --- | --- | --- | --- | --- | --- | --- |
|  | **Size (Mb)** | **Total SNPs** | **Distance between SNPs (kb)** | **Size (Mb)** | **Total SNPs** | **Distance between SNPs (kb)** |
| 1 | 36.50 | 1,695 | 21.5 | 36.39 | 1,177 | 30.9 |
| 2 | 25.36 | 1,491 | 17.0 | 25.07 | 778 | 32.2 |
| 3 | 12.95 | 1,095 | 11.8 | 12.87 | 613 | 21.0 |
| 4 | 20.81 | 1,113 | 18.7 | 20.54 | 663 | 30.9 |
| 5 | 37.18 | 2,253 | 16.5 | 37.05 | 975 | 38.0 |
| 6 | 37.44 | 1,806 | 20.7 | 37.29 | 1,143 | 32.6 |
| 7 | 55.60 | 2,716 | 20.4 | 55.50 | 1,255 | 44.2 |
| 8 | 45.73 | 2,584 | 17.7 | 45.30 | 1,461 | 31.0 |
| 9 | 21.01 | 1,268 | 16.5 | 20.98 | 829 | 25.3 |
| 10 | 21.00 | 1,243 | 16.8 | 20.92 | 634 | 33.0 |
| 11 | 19.73 | 1,276 | 15.4 | 19.49 | 696 | 28.0 |
| Contigs | 68.6 | 4,304 | 15.9 | – | – |  |
| Total | 401.9 | 22,844 | 208.9 | 331.4 | 10,224 | 347.1 |
| Average | 36.5 | 2,076.7 | 17.4 | 30.13 | 929.4 | 31.5 |

**Supplementary Table 5| Degrees of freedom (df), F values and probabilities of analysis of variance for cohort, check genotypes, treatments, and their interactions in mungbean** mini−core **collection genotypes screened in two cohorts at the germination stage.**

| **Trait** | **Source of Variation** | **Cohort** | **Treat** | **Gen** | **Gen × Cohort** | **Treat × Cohort** | **Treat × Gen** | **Treat × Gen × Cohort** | **Treat × Gen/**  **Treat × Gen × Cohort*** |
| --- | --- | --- | --- | --- | --- | --- | --- | --- | --- |
|  | df | 1 | 1 | 7 | 7 | 1 | 7 | 7 | 7/7 |
| Emergence (%) | F value | 7.12 | 3450.33 | 47.51 | 17.65 | 3.09 | 32.41 | 4.03 | 8.04 |
|  | Probability | 0.11 | <.001 | <.001 | 0.01 | 0.01 | <.001 | 0.001 | 0.01 |
| Shoot dry mass (g) | F value | 10.58 | 1789.09 | 54.81 | 8.55 | 4.02 | 28.78 | 2.93 | 9.82 |
|  | Probability | 0.08 | <.001 | <.001 | 0.04 | 0.001 | <.001 | 0.011 | 0.004 |
| Root dry mass (g) | F value | 51.47 | 4265.91 | 39.31 | 18.05 | 3.05 | 14.48 | 2.63 | 5.05 |
|  | Probability | 0.02 | <.001 | <.001 | 0.01 | 0.01 | <.001 | 0.02 | 0.02 |
| Total dry mass (g) | F value | 14.95 | 2272.43 | 68.06 | 10.39 | 5.06 | 31.97 | 3.85 | 8.30 |
|  | Probability | 0.06 | <.001 | <.001 | 0.04 | <.001 | <.001 | 0.002 | 0.01 |

*Note: Further analysis (Treat × Gen/Treat × Gen × Cohort) was undertaken to understand whether the genotype response to waterlogging was greater than the cohort effect for this interaction.

Supplementary Table 6| Summary statistics for mungbean mini−core collection genotypes screened at the germination and seedling stages under control and waterlogging (WL) conditions. Plants WL for 4 days at the germination stage to measure emergence (%) and shoot, root and total dry mass, and 8 days at the seedling stage to measure shoot, root and total dry mass and SPAD chlorophyll content.

| **Variable** | **Mean** | | **Minimum** | | **Maximum** | | **SD** | | **Skewness** | | **Kurtosis** | | **Broad-sense heritability (%)** |
| --- | --- | --- | --- | --- | --- | --- | --- | --- | --- | --- | --- | --- | --- |
|  | **Control** | **WL** | **Control** | **WL** | **Control** | **WL** | **Control** | **WL** | **Control** | **WL** | **Control** | **WL** |  |
| **Germination stage** |  |  |  |  |  |  |  |  |  |  |  |  |  |
| Emergence (%) | 96 | 48 | 91 | 0.0 | 100 | 90 | 0.6 | 1.2 | –0.3 | –0.2 | –1.1 | –0.6 | 81 |
| Shoot dry mass (g) | 0.4 | 0.2 | 0.1 | 0.0 | 0.9 | 0.1 | 0.3 | 0.1 | 0.2 | 1.0 | –0.3 | 0.9 | 83 |
| Root dry mass (g) | 0.3 | 0.1 | 0.1 | 0.0 | 0.6 | 0.4 | 0.1 | 0.1 | 1.8 | 1.2 | 5.2 | 2.4 | 71 |
| Total dry mass (g) | 0.9 | 0.2 | 0.3 | 0.0 | 2.3 | 1.1 | 0.4 | 0.2 | 0.4 | 1.0 | 0.0 | 1.0 | 83 |
| **Seedling stage** |  |  |  |  |  |  |  |  |  |  |  |  |  |
| Total dry mass (g) | 1.2 | 0.6 | 0.4 | 0.2 | 2.8 | 1.5 | 0.4 | 0.2 | 0.8 | 0.9 | 0.8 | 1.1 | 79 |
| Shoot dry mass (g) | 0.8 | 0.4 | 0.2 | 0.1 | 1.8 | 1.0 | 0.3 | 0.2 | 0.7 | 0.9 | 0.4 | 0.9 | 81 |
| Root dry mass (g) | 0.4 | 0.2 | 0.1 | 0.02 | 1.1 | 0.6 | 0.2 | 0.1 | 1.1 | 1.1 | 1.7 | 2.3 | 78 |
| Adventitious root number | 0.0 | 12 | 0.0 | 0.0 | 0.0 | 25 | 0.0 | 4.9 |  | 0.1 |  | 0.1 | 56 |
| SPAD chlorophyll content | 38.4 | 27.1 | 31.0 | 15.1 | 46.9 | 37.8 | 2.6 | 3.9 | 0.3 | –0.1 | 0.2 | 0.4 | 70 |

Supplementary Table 7| Principal component loadings of germination traits [emergence (%) and shoot, root and total dry mass] and seedling traits [shoot, root and total dry mass, adventitious root number, SPAD chlorophyll content] in the mungbean mini core population showing Eigenvalues and contributions of PC1, PC2 and PC3 to total variance and cumulative (%). The data were normalised for analysis.

| **Growth stage** | **Waterlogging** | | |
| --- | --- | --- | --- |
|  | **PC1** | **PC2** | **PC3** |
| **Germination stage** |  |  |  |
| Emergence (%) | 0.39 | –0.32 | 0.85 |
| Shoot dry mass (g) | **0.54** | 0.05 | –0.13 |
| Root dry mass (g) | **0.50** | 0.07 | –0.33 |
| Total dry mass (g) | **0.55** | 0.05 | –0.20 |
| Eigenvalues | 3.14 | 1.05 | 0.57 |
| Variability (%) | 63.00 | 21.00 | 11.34 |
| Cumulative (%) |  |  | 95.34 |
|  |  |  |  |
| **Seedling stage** |  |  |  |
| Shoot dry mass (g) | 0.013 | 0.018 | 0.004 |
| Root dry mass (g) | 0.010 | 0.007 | –0.002 |
| Total dry mass (g) | 0.023 | 0.025 | 0.002 |
| Adventitious root number | **0.620** | **0.761** | 0.153 |
| SPAD chlorophyll content | –**0.688** | 0.433 | **0.573** |
| Eigenvalues | 19.92 | 15.24 | 8.78 |
| Variability (%) | 39.48 | 30.2 | 17.41 |
| Cumulative (%) |  |  | 87.09 |

**Supplementary Table 8| Analysis of variance (ANOVA) based on the origin of the mungbean** mini−core **collection genotypes screened at the germination and seedling stages under control and waterlogging (WL) conditions. Plants WL for 4 days at the germination stage to measure emergence (%) and shoot, root and total dry mass, and 8 days at the seedling stage to measure shoot, root and total dry mass and SPAD chlorophyll content.**

| **Source of variation** | **Germination stage** | | | | **Seedling stage** | | | | | | |
| --- | --- | --- | --- | --- | --- | --- | --- | --- | --- | --- | --- |
|  | **Emergence (%)** | **Shoot dry mass (g)** | **Root dry mass (g)** | **Total dry mass (g)** | **Shoot dry mass (g)** | **Root dry mass (g)** | **Total dry mass (g)** | **Adventitious root number** | **SPAD unit_1** | **SPAD unit_2** | **SPAD unit_3** |
| df | 9 | 9 | 9 | 9 | 9 | 9 | 9 | 9 | 9 | 9 | 9 |
| F value | 4.19 | 2.32 | 1.72 | 2.26 | 9.60 | 11.88 | 11.25 | 2.88 | 2.20 | 1.04 | 0.60 |
| Probability | <0.001 | 0.01 | 0.08 | 0.02 | <.001 | <.001 | <.001 | 0.002 | 0.02 | 0.40 | 0.80 |

Supplementary Table 9| Degrees of freedom (df), F values and probabilities of analysis of variance for cohort, check genotypes (Gen), treatments (Treat), and their interactions in mungbean mini−core collection genotypes in three cohorts screened at the seedling stage.

| **Trait** | **Source of variation** | **Cohort** | **Gen** | **Treat** | **Gen × Cohort** | **Treat × Cohort** | **Treat × Gen** | **Treat × Gen × Cohort** | **Treat × Gen/**  **Treat × Gen × Cohort** |
| --- | --- | --- | --- | --- | --- | --- | --- | --- | --- |
|  | df | 2 | 7 | 1 | 14 | 2 | 7 | 14 | 7/14 |
| Total dry mass (g) | F value | 32.1 | 13.43 | 122.21 | 0.98 | 6.55 | 2.35 | 0.91 | 2.58 |
|  | Probability | 0.003 | <0.001 | <0.001 | 0.48 | 0.03 | 0.03 | 0.55 | 0.06 |
| Shoot dry mass (g) | F value | 24.02 | 10.81 | 111.26 | 0.68 | 5.34 | 2.58 | 0.68 | 3.76 |
|  | Probability | 0.01 | <0.001 | <0.001 | 0.79 | 0.05 | 0.02 | 0.78 | 0.02 |
| Root dry mass (g) | F value | 26.94 | 14.1 | 110.08 | 2.42 | 6.69 | 2.12 | 1.11 | 1.92 |
|  | Probability | 0.01 | <0.001 | <0.001 | 0.01 | 0.03 | 0.05 | 0.36 | 0.14 |
| Adventitious root number | F value | 19.09 | 6.27 | 2420.66 | 1.09 | 18.99 | 6.27 | 1.09 | 5.75 |
|  | Probability | 0.01 | <0.001 | <0.001 | 0.378 | 0.003 | <0.001 | 0.378 | 0.002 |
| SPAD chlorophyll content | F value | 44.36 | 7.05 | 301.01 | 3.12 | 4.96 | 2.85 | 2.14 | 0.67 |
|  | Probability | 0.002 | <0.001 | <0.001 | <0.001 | 0.05 | 0.01 | 0.02 | 0.69 |

*Note: Further analysis (Treat × Gen/Treat × Gen × Cohort) was undertaken to understand whether the genotype response to waterlogging was greater than the cohort effect for this interaction.

**Supplementary Table 10| List of possible candidate genes within the estimated LD decay distance for the formation of adventitious roots based on NCBI (www.ncbi.nlm.nih.gov).**

| **Gene ID** | **Position (bp)** | **Transcript** | **Length (nt)** | **Protein** | **Length (aa)** | **Protein name** | **Isoform** | ***Arabidopsis thaliana*** |
| --- | --- | --- | --- | --- | --- | --- | --- | --- |
| LOC106765473 | 45,074,500 | XM_014650115.2 | 2168 | XP_014505601.1 | 516 | AAA-ATPase At5g57480 |  | *At5g57480* |
| LOC106765510 | 45,055,664 | XM_014650165.2 | 1172 | XP_014505651.1 | 269 | protein YIPF6 homolog |  |  |
| LOC106765516 | 45,047,237 | XM_014650171.2 | 1370 | XP_014505657.1 | 277 | mitochondrial outer membrane protein porin 6 |  |  |
| LOC106766492 | 45,038,362 | XM_014651216.2 | 1106 | XP_014506702.1 | 321 | beta-1,3-galactosyltransferase 6 |  | *At1g26810* |
| LOC106767325 | 45,018,523 | XM_014652187.2 | 2171 | XP_014507673.1 | 587 | 4-coumarate--CoA ligase 2 |  | *At3g21240* |
| LOC106767575 | 45,009,156 | XM_014652497.2 | 1999 | XP_014507983.1 | 539 | 4-coumarate--CoA ligase 2-like |  | *At3g21240* |
| LOC106768043 | 44,984,591 | XM_014653009.2 | 1357 | XP_014508495.2 | 239 | zinc finger protein 3 |  | *At5g43170* |
| LOC106767765 | 44,972,496 | XM_014652716.2 | 749 | XP_014508202.1 | 180 | CASP-like protein 2C1 |  | *At4g25830* |
| LOC106766748 | 44,962,987 | XM_014651489.2 | 1090 | XP_014506975.1 | 292 | probable xyloglucan endotransglucosylase/hydrolase protein 23 |  | *At4g25810* |
| LOC106769017 | 44,956,059 | XM_014654456.2 | 1023 | XP_014509942.1 | 284 | probable xyloglucan endotransglucosylase/hydrolase protein 23 |  | *At4g25811* |
| LOC106768744 | 44,952,950 | XM_014654033.2 | 1215 | XP_014509519.1 | 284 | probable xyloglucan endotransglucosylase/hydrolase protein 23 |  | *At4g25812* |
| LOC106768890 | 44,950,068 | XM_014654263.2 | 1183 | XP_014509749.1 | 291 | xyloglucan endotransglucosylase/hydrolase protein 22 |  | *At5g57560* |
| LOC106768904 | 44,947,172 | XM_014654281.2 | 1148 | XP_014509767.1 | 284 | xyloglucan endotransglucosylase/hydrolase protein 22 |  | *At5g57561* |
| LOC106768551 | 44,942,506 | XM_022783887.1 | 2619 | XP_022639608.1 | 635 | calmodulin-binding protein 60 B |  | *At5g57580* |
| LOC106768551 | 44,942,534 | XM_022783888.1 | 2585 | XP_022639609.1 | 635 | calmodulin-binding protein 60 B |  |  |
| LOC106768551 |  | XM_014653768.2 | 2575 | XP_014509254.1 | 635 | calmodulin-binding protein 60 B |  |  |
| LOC106768551 |  | XM_014653769.2 | 2515 | XP_014509255.1 | 635 | calmodulin-binding protein 60 B |  |  |
| LOC106768551 |  | XM_022783889.1 | 2565 | XP_022639610.1 | 635 | calmodulin-binding protein 60 B |  |  |
| LOC106768551 |  | XM_014653767.2 | 2608 | XP_014509253.1 | 635 | calmodulin-binding protein 60 B |  |  |
| LOC106768551 |  | XM_014653770.2 | 2554 | XP_014509256.1 | 635 | calmodulin-binding protein 60 B |  |  |
| LOC106769165 | 44,932,120 | XM_014654667.2 | 3162 | XP_014510153.1 | 820 | bifunctional dethiobiotin synthetase/7,8-diamino-pelargonic acid aminotransferase, mitochondrial | X1 | *At5g57590* |
| LOC106769165 |  | XM_014654668.2 | 3154 | XP_014510154.1 | 818 | bifunctional dethiobiotin synthetase/7,8-diamino-pelargonic acid aminotransferase, mitochondrial | X2 |  |
| LOC106768395 | 44,902,960 | XM_014653538.2 | 4798 | XP_014509024.1 | 1117 | angiopoietin-1 receptor | X1 |  |
| LOC106768395 |  | XM_022783884.1 | 4053 | XP_022639605.1 | 1036 | uncharacterized protein LOC106768395 | X2 |  |
| LOC106767894 | 44,893,748 | XM_014652857.2 | 1608 | XP_014508343.1 | 361 | transcription factor MYB36 |  | *At5g57620* |
| LOC106766491 | 44,888,795 | XM_014651215.2 | 1729 | XP_014506701.1 | 492 | flavonoid 3-O-glucosyltransferase |  | *At5g17050* |
| LOC106769348 | 44,872,227 | XM_014654941.2 | 1694 | XP_014510427.1 | 468 | CBL-interacting serine/threonine-protein kinase 21 |  | *At5g57630* |
| LOC106769348 |  | XR_002668815.1 | 1755 |  |  |  |  |  |
| LOC106769348 |  | XM_022783882.1 | 1672 | XP_022639603.1 | 468 | CBL-interacting serine/threonine-protein kinase 21 |  |  |
| LOC106769348 |  | XM_022783881.1 | 1978 | XP_022639602.1 | 468 | CBL-interacting serine/threonine-protein kinase 21 |  |  |
| LOC106767356 | 44,877,524 | XM_014652228.2 | 951 | XP_014507714.1 | 173 | pathogenesis-related protein PR-1 |  | *At2g14610* |
| LOC106769349 | 44,874,837 | XM_014654943.2 | 837 | XP_014510429.1 | 200 | pathogenesis-related protein PR-1-like |  | *At2g14610* |
| LOC106765461 | 44,859,229 | XM_014650096.2 | 1758 | XP_014505582.1 | 479 | xylose isomerase |  | *At5g57655* |
| LOC106765461 |  | XM_014650097.2 | 1736 | XP_014505583.1 | 479 | xylose isomerase |  |  |
| LOC106765461 |  | XM_022783880.1 | 1683 | XP_022639601.1 | 479 | xylose isomerase |  |  |
| LOC106768460 | 44,850,692 | XM_014653635.2 | 1593 | XP_014509121.1 | 182 | ADP-ribosylation factor 1 |  | *At2g47170* |
| LOC106768460 |  | XM_014653634.2 | 1715 | XP_014509120.1 | 182 | ADP-ribosylation factor 1 |  |  |
| LOC106769383 | 44,824,150 | XM_014654989.2 | 2323 | XP_014510475.1 | 731 | 1-deoxy-D-xylulose-5-phosphate synthase, chloroplastic |  | *At4g15560* |
| LOC106769252 | 44,802,115 | XM_014654789.2 | 1686 | XP_014510275.1 | 372 | zinc finger protein CONSTANS-LIKE 5 |  | *At5g57660* |
| LOC106767602 | 44,783,732 | XM_022782968.1 | 2489 | XP_022638689.1 | 606 | probable receptor-like serine/threonine-protein kinase At5g57670 | X1 | *At5g57670* |
| LOC106767602 |  | XM_014652525.2 | 2485 | XP_014508011.1 | 605 | probable receptor-like serine/threonine-protein kinase At5g57670 | X2 |  |
| LOC106766490 | 44,777,857 | XM_014651214.2 | 1184 | XP_014506700.1 | 380 | PTI1-like tyrosine-protein kinase At3g15890 |  | *At3g15890* |
| LOC106766955 | 44,769,595 | XM_014651756.2 | 857 | XP_014507242.1 | 164 | protein GLUTAMINE DUMPER 2 |  | *At5g57685* |
| LOC106767522 | 44,738,932 | XM_014652436.2 | 610 | XP_014507922.1 | 88 | small nuclear ribonucleoprotein E |  | *At2g18740* |

Supplementary Table 11| List of Possible candidate genes within the estimated LD decay distance for the SPAD chlorophyll content on the first trifoliate leaves based on NCBI ((www.ncbi.nlm.nih.gov).

| **Gene ID** | **Position (bp)** | **Transcript** | **Length (nt)** | **Protein** | **Length (aa)** | **Protein name** | **Isoform** | ***Arabidopsis thaliana*** |
| --- | --- | --- | --- | --- | --- | --- | --- | --- |
| LOC106770352 | 20,709,746 | XM_014656166.1 | 2331 | XP_014511652.1 | 776 | protein FAR1-RELATED SEQUENCE 5-like |  | *At4g38180* |
| LOC106769646 | 20,751,509 | XM_014655352.2 | 1832 | XP_014510838.1 | 397 | cleavage stimulating factor 64 | X1 | *At1g71800* |
| LOC106769646 |  | XM_014655353.2 | 1869 | XP_014510839.1 | 395 | cleavage stimulating factor 64 | X2 |  |
| LOC106770951 | 20,760,356 | XM_014656808.2 | 1576 | XP_014512294.1 | 360 | tetratricopeptide repeat protein 4 homolog |  | *At1g04130* |
| LOC106771196 | 20,764,961 | XM_014657124.2 | 1540 | XP_014512610.1 | 379 | alcohol dehydrogenase class-3 | X2 | *At5g43940* |
| LOC106771196 |  | XM_022784887.1 | 1576 | XP_022640608.1 | 391 | alcohol dehydrogenase class-3 | X1 |  |
| LOC106770356 | 20,821,742 | XM_014656169.1 | 2142 | XP_014511655.1 | 713 | protein FAR-RED IMPAIRED RESPONSE 1-like |  | *At4g15090* |

**Supplementary Table 12| Genotype means from screening of 292 mungbean** mini−core **genotypes for waterlogging tolerance at the germination stage. The treatment was waterlogged for 4 days (WL 4 days) and compared with their respective drained control. The waterlogged pots recovered for 7 days immediately after the release of waterlogging. Harvesting of waterlogged and control pots occurred on the same day. Data represent the mean value for three replications.**

| **Sr. No.** | **Accession Num** | **Origin** | **Germination stage** | | | | | | | |
| --- | --- | --- | --- | --- | --- | --- | --- | --- | --- | --- |
|  |  |  | **Emergence (%)** | | **Shoot dry mass (g)** | | **Root dry mass (g)** | | **Total dry mass (g)** | |
|  |  |  | **Control** | **WL** | **Control** | **WL** | **Control** | **WL** | **Control** | **WL** |
| 1 | AGG325466 | SEA | 100 | 53 | 0.40 | 0.29 | 0.21 | 0.13 | 0.61 | 0.43 |
| 2 | AGG325467 | SA | 100 | 75 | 0.53 | 0.42 | 0.41 | 0.06 | 0.94 | 0.49 |
| 3 | AGG325468 | SA | 97 | 20 | 1.13 | 0.09 | 0.48 | 0.05 | 1.61 | 0.15 |
| 4 | AGG325469 | SWA | 100 | 32 | 0.26 | 0.06 | 0.14 | 0.03 | 0.40 | 0.10 |
| 5 | AGG325470 | SWA | 100 | 39 | 0.97 | 0.06 | 0.37 | 0.04 | 1.34 | 0.11 |
| 6 | AGG325471 | SA | 100 | 27 | 0.77 | 0.16 | 0.32 | 0.07 | 1.09 | 0.25 |
| 7 | AGG325472 | SWA | 100 | 29 | 0.69 | 0.08 | 0.25 | 0.05 | 0.94 | 0.15 |
| 8 | AGG325473 | SWA | 94 | 30 | 1.09 | 0.05 | 0.38 | 0.02 | 1.47 | 0.08 |
| 9 | AGG325474 | NA | 100 | 56 | 0.53 | 0.14 | 0.28 | 0.08 | 0.81 | 0.23 |
| 10 | AGG325475 | SWA | 92 | 36 | 1.23 | 0.15 | 0.46 | 0.08 | 1.69 | 0.24 |
| 11 | AGG325476 | SWA | 96 | 37 | 0.71 | 0.12 | 0.26 | 0.07 | 0.97 | 0.21 |
| 12 | AGG325477 | SA | 93 | 46 | 0.60 | 0.19 | 0.24 | 0.09 | 0.84 | 0.29 |
| 13 | AGG325478 | SWA | 96 | 70 | 0.64 | 0.09 | 0.20 | 0.05 | 0.84 | 0.16 |
| 14 | AGG325479 | SWA | 99 | 62 | 0.53 | 0.07 | 0.20 | 0.05 | 0.72 | 0.13 |
| 15 | AGG325480 | SWA | 94 | 31 | 0.73 | 0.04 | 0.23 | 0.03 | 0.96 | 0.08 |
| 16 | AGG325481 | SEA | 98 | 49 | 0.98 | 0.13 | 0.32 | 0.03 | 1.31 | 0.17 |
| 17 | AGG325482 | SEA | 94 | 18 | 0.76 | 0.04 | 0.25 | 0.02 | 1.01 | 0.07 |
| 18 | AGG325483 | SWA | 100 | 56 | 0.38 | 0.11 | 0.26 | 0.04 | 0.63 | 0.16 |
| 19 | AGG325484 | SA | 94 | 49 | 0.62 | 0.18 | 0.29 | 0.07 | 0.90 | 0.27 |
| 20 | AGG325485 | SA | 95 | 66 | 0.49 | 0.19 | 0.20 | 0.06 | 0.68 | 0.26 |
| 21 | AGG325486 | SA | 100 | 31 | 0.71 | 0.21 | 0.20 | 0.05 | 0.91 | 0.27 |
| 22 | AGG325487 | SA | 100 | 70 | 0.36 | 0.08 | 0.34 | 0.04 | 0.70 | 0.13 |
| 23 | AGG325488 | SA | 100 | 49 | 0.47 | 0.10 | 0.21 | 0.05 | 0.68 | 0.17 |
| 24 | AGG325489 | SA | 100 | 56 | 0.37 | 0.04 | 0.19 | 0.04 | 0.56 | 0.10 |
| 25 | AGG325490 | SA | 99 | 66 | 0.51 | 0.13 | 0.61 | 0.06 | 1.12 | 0.20 |
| 26 | AGG325491 | SA | 98 | 0 | 1.21 | 0.00 | 0.68 | 0.00 | 1.89 | 0.00 |
| 27 | AGG325492 | SAM | 95 | 35 | 0.68 | 0.10 | 0.31 | 0.06 | 0.99 | 0.16 |
| 28 | AGG325493 | SA | 95 | 4 | 0.78 | 0.01 | 0.47 | 0.02 | 1.26 | 0.04 |
| 29 | AGG325494 | SA | 94 | 30 | 0.51 | 0.13 | 0.32 | 0.08 | 0.83 | 0.22 |
| 30 | AGG325495 | NA | 94 | 47 | 0.60 | 0.26 | 0.24 | 0.13 | 0.84 | 0.40 |
| 31 | AGG325496 | SWA | 96 | 53 | 0.60 | 0.08 | 0.18 | 0.05 | 0.79 | 0.14 |
| 32 | AGG325497 | SA | 94 | 34 | 0.55 | 0.09 | 0.35 | 0.05 | 0.90 | 0.15 |
| 33 | AGG325498 | SA | 100 | 30 | 0.43 | 0.03 | 0.21 | 0.02 | 0.64 | 0.06 |
| 34 | AGG325499 | SA | 100 | 62 | 0.64 | 0.25 | 0.27 | 0.14 | 0.91 | 0.39 |
| 35 | AGG325500 | SA | 97 | 60 | 0.70 | 0.18 | 0.17 | 0.05 | 0.87 | 0.25 |
| 36 | AGG325501 | SA | 99 | 21 | 0.55 | 0.04 | 0.25 | 0.02 | 0.79 | 0.07 |
| 37 | AGG325502 | SA | 95 | 54 | 0.61 | 0.16 | 0.17 | 0.13 | 0.78 | 0.30 |
| 38 | AGG325503 | SA | 92 | 51 | 1.20 | 0.28 | 0.54 | 0.14 | 1.74 | 0.43 |
| 39 | AGG325504 | SA | 94 | 74 | 0.89 | 0.26 | 0.28 | 0.15 | 1.17 | 0.42 |
| 40 | AGG325505 | SA | 94 | 29 | 1.12 | 0.08 | 0.48 | 0.06 | 1.60 | 0.15 |
| 41 | AGG325506 | SA | 94 | 44 | 0.85 | 0.35 | 0.38 | 0.15 | 1.23 | 0.51 |
| 42 | AGG325507 | SA | 94 | 72 | 0.84 | 0.43 | 0.23 | 0.17 | 1.07 | 0.61 |
| 43 | AGG325508 | SA | 92 | 48 | 1.11 | 0.21 | 0.34 | 0.04 | 1.45 | 0.27 |
| 44 | AGG325509 | SA | 100 | 50 | 1.49 | 0.25 | 0.77 | 0.12 | 2.26 | 0.39 |
| 45 | AGG325510 | SEA | 95 | 75 | 0.95 | 0.50 | 0.30 | 0.15 | 1.25 | 0.66 |
| 46 | AGG325511 | SA | 94 | 31 | 1.05 | 0.09 | 0.37 | 0.06 | 1.41 | 0.16 |
| 47 | AGG325512 | OP | 97 | 46 | 0.31 | 0.19 | 0.21 | 0.02 | 0.52 | 0.23 |
| 48 | AGG325513 | OP | 97 | 56 | 1.33 | 0.37 | 0.45 | 0.13 | 1.77 | 0.51 |
| 49 | AGG325514 | OP | 97 | 65 | 1.67 | 0.36 | 0.55 | 0.16 | 2.22 | 0.53 |
| 50 | AGG325515 | OP | 95 | 15 | 0.81 | 0.05 | 0.50 | 0.02 | 1.31 | 0.08 |
| 51 | AGG325516 | OP | 92 | 64 | 0.61 | 0.08 | 0.24 | 0.03 | 0.85 | 0.12 |
| 52 | AGG325517 | SEA | 97 | 71 | 0.93 | 0.26 | 0.36 | 0.08 | 1.29 | 0.35 |
| 53 | AGG325518 | SEA | 92 | 32 | 0.96 | 0.10 | 0.27 | 0.05 | 1.24 | 0.16 |
| 54 | AGG325519 | SEA | 95 | 49 | 1.23 | 0.15 | 0.56 | 0.07 | 1.78 | 0.23 |
| 55 | AGG325520 | SEA | 96 | 16 | 1.41 | 0.09 | 0.39 | 0.04 | 1.80 | 0.14 |
| 56 | AGG325521 | SA | 94 | 49 | 0.70 | 0.13 | 0.32 | 0.07 | 1.02 | 0.20 |
| 57 | AGG325522 | SA | 95 | 61 | 0.61 | 0.09 | 0.19 | 0.05 | 0.80 | 0.14 |
| 58 | AGG325523 | SA | 100 | 90 | 0.79 | 0.17 | 0.25 | 0.07 | 1.05 | 0.25 |
| 59 | AGG325524 | SEA | 92 | 55 | 1.14 | 0.38 | 0.45 | 0.07 | 1.59 | 0.46 |
| 60 | AGG325525 | SA | 93 | 41 | 0.61 | 0.15 | 0.20 | 0.08 | 0.80 | 0.25 |
| 61 | AGG325526 | SA | 92 | 49 | 0.98 | 0.13 | 0.23 | 0.16 | 1.22 | 0.30 |
| 62 | AGG325527 | SA | 97 | 65 | 0.67 | 0.29 | 0.26 | 0.14 | 0.94 | 0.45 |
| 63 | AGG325528 | SWA | 96 | 0 | 0.39 | 0.00 | 0.14 | 0.00 | 0.53 | 0.00 |
| 64 | AGG325529 | SA | 92 | 45 | 0.46 | 0.11 | 0.14 | 0.03 | 0.60 | 0.15 |
| 65 | AGG325530 | SA | 100 | 76 | 0.75 | 0.23 | 0.25 | 0.08 | 1.00 | 0.32 |
| 66 | AGG325531 | SA | 95 | 56 | 0.72 | 0.19 | 0.24 | 0.08 | 0.96 | 0.28 |
| 67 | AGG325532 | SA | 93 | 42 | 0.72 | 0.19 | 0.20 | 0.06 | 0.93 | 0.27 |
| 68 | AGG325533 | NA | 92 | 48 | 0.68 | 0.19 | 0.19 | 0.07 | 0.87 | 0.26 |
| 69 | AGG325534 | SA | 100 | 51 | 0.41 | 0.02 | 0.12 | 0.02 | 0.53 | 0.05 |
| 70 | AGG325535 | SA | 93 | 67 | 1.33 | 0.30 | 0.41 | 0.12 | 1.73 | 0.43 |
| 71 | AGG325536 | SA | 94 | 33 | 0.84 | 0.21 | 0.23 | 0.14 | 1.07 | 0.36 |
| 72 | AGG325537 | SWA | 100 | 15 | 0.80 | 0.02 | 0.23 | 0.02 | 1.03 | 0.05 |
| 73 | AGG325538 | SWA | 100 | 45 | 0.76 | 0.13 | 0.21 | 0.08 | 0.97 | 0.21 |
| 74 | AGG325539 | SA | 100 | 56 | 0.92 | 0.15 | 0.30 | 0.05 | 1.22 | 0.21 |
| 75 | AGG325540 | SA | 100 | 51 | 0.47 | 0.15 | 0.15 | 0.10 | 0.62 | 0.26 |
| 76 | AGG325541 | SA | 100 | 82 | 0.39 | 0.14 | 0.15 | 0.07 | 0.54 | 0.23 |
| 77 | AGG325542 | SA | 100 | 18 | 0.88 | 0.06 | 0.39 | 0.01 | 1.26 | 0.08 |
| 78 | AGG325543 | SA | 95 | 29 | 0.60 | 0.06 | 0.17 | 0.03 | 0.76 | 0.10 |
| 79 | AGG325544 | SA | 97 | 53 | 0.70 | 0.19 | 0.22 | 0.08 | 0.92 | 0.28 |
| 80 | AGG325545 | SA | 100 | 39 | 1.16 | 0.09 | 0.15 | 0.06 | 1.31 | 0.15 |
| 81 | AGG325546 | NA | 100 | 51 | 0.29 | 0.14 | 0.12 | 0.08 | 0.41 | 0.24 |
| 82 | AGG325547 | SA | 100 | 75 | 0.32 | 0.13 | 0.18 | 0.06 | 0.50 | 0.20 |
| 83 | AGG325548 | SA | 95 | 73 | 0.44 | 0.13 | 0.19 | 0.09 | 0.63 | 0.23 |
| 84 | AGG325549 | SA | 95 | 45 | 0.77 | 0.11 | 0.30 | 0.05 | 1.07 | 0.17 |
| 85 | AGG325550 | SA | 97 | 42 | 1.01 | 0.18 | 0.50 | 0.07 | 1.51 | 0.26 |
| 86 | AGG325551 | UK | 100 | 53 | 0.38 | 0.13 | 0.13 | 0.04 | 0.51 | 0.18 |
| 87 | AGG325552 | SA | 100 | 30 | 1.06 | 0.10 | 0.87 | 0.05 | 1.93 | 0.16 |
| 88 | AGG325553 | SA | 100 | 40 | 0.47 | 0.08 | 0.29 | 0.05 | 0.76 | 0.14 |
| 89 | AGG325554 | SA | 100 | 31 | 0.77 | 0.09 | 0.25 | 0.04 | 1.02 | 0.14 |
| 90 | AGG325555 | SA | 98 | 62 | 1.52 | 0.14 | 0.61 | 0.08 | 2.14 | 0.23 |
| 91 | AGG325556 | SA | 94 | 70 | 1.05 | 0.20 | 0.41 | 0.09 | 1.47 | 0.29 |
| 92 | AGG325557 | SA | 96 | 32 | 0.70 | 0.05 | 0.19 | 0.02 | 0.89 | 0.08 |
| 93 | AGG325558 | SA | 100 | 65 | 1.01 | 0.22 | 0.33 | 0.07 | 1.34 | 0.29 |
| 94 | AGG325559 | SA | 100 | 52 | 0.53 | 0.11 | 0.19 | 0.06 | 0.72 | 0.19 |
| 95 | AGG325560 | SA | 96 | 35 | 0.99 | 0.13 | 0.37 | 0.05 | 1.37 | 0.18 |
| 96 | AGG325561 | SA | 94 | 38 | 0.78 | 0.22 | 0.34 | 0.14 | 1.12 | 0.37 |
| 97 | AGG325562 | SA | 99 | 23 | 0.97 | 0.09 | 0.26 | 0.04 | 1.22 | 0.14 |
| 98 | AGG325563 | SA | 94 | 50 | 1.00 | 0.15 | 0.39 | 0.08 | 1.39 | 0.24 |
| 99 | AGG325564 | SWA | 94 | 49 | 0.97 | 0.21 | 0.35 | 0.08 | 1.32 | 0.30 |
| 100 | AGG325565 | SWA | 99 | 9 | 0.96 | 0.03 | 0.28 | 0.03 | 1.24 | 0.06 |
| 101 | AGG325566 | EUR | 100 | 25 | 0.96 | 0.09 | 0.37 | 0.05 | 1.33 | 0.14 |
| 102 | AGG325567 | SEA | 100 | 57 | 1.41 | 0.35 | 0.49 | 0.18 | 1.90 | 0.54 |
| 103 | AGG325568 | EA | 100 | 86 | 1.08 | 0.39 | 0.34 | 0.18 | 1.42 | 0.59 |
| 104 | AGG325569 | EA | 93 | 0 | 0.73 | 0.00 | 0.21 | 0.00 | 0.94 | 0.00 |
| 105 | AGG325570 | SA | 96 | 65 | 1.17 | 0.14 | 0.81 | 0.06 | 1.98 | 0.21 |
| 106 | AGG325571 | SA | 100 | 22 | 0.58 | 0.38 | 0.25 | 0.09 | 0.83 | 0.48 |
| 107 | AGG325572 | SA | 94 | 53 | 0.58 | 0.28 | 0.22 | 0.16 | 0.79 | 0.46 |
| 108 | AGG325573 | NA | 100 | 63 | 0.96 | 0.38 | 0.25 | 0.16 | 1.21 | 0.55 |
| 109 | AGG325574 | SA | 91 | 33 | 0.72 | 0.16 | 0.25 | 0.06 | 0.97 | 0.22 |
| 110 | AGG325575 | SA | 93 | 82 | 1.03 | 0.45 | 0.35 | 0.21 | 1.37 | 0.66 |
| 111 | AGG325576 | SA | 95 | 55 | 1.08 | 0.12 | 0.35 | 0.09 | 1.43 | 0.23 |
| 112 | AGG325577 | SA | 93 | 10 | 0.50 | 0.02 | 0.21 | 0.02 | 0.71 | 0.05 |
| 113 | AGG325578 | SA | 95 | 80 | 0.76 | 0.31 | 0.49 | 0.15 | 1.25 | 0.47 |
| 114 | AGG325579 | SEA | 92 | 60 | 1.20 | 0.38 | 0.51 | 0.12 | 1.70 | 0.51 |
| 115 | AGG325580 | EA | 100 | 16 | 1.01 | 0.04 | 0.22 | 0.02 | 1.23 | 0.07 |
| 116 | AGG325581 | SEA | 92 | 51 | 0.92 | 0.21 | 0.23 | 0.10 | 1.15 | 0.32 |
| 117 | AGG325582 | SWA | 96 | 61 | 0.70 | 0.07 | 0.26 | 0.06 | 0.95 | 0.14 |
| 118 | AGG325584 | SWA | 94 | 11 | 0.56 | 0.10 | 0.19 | 0.02 | 0.76 | 0.14 |
| 119 | AGG325585 | SEA | 96 | 53 | 0.76 | 0.07 | 0.32 | 0.06 | 1.09 | 0.14 |
| 120 | AGG325586 | SEA | 100 | 39 | 0.88 | 0.33 | 0.26 | 0.09 | 1.14 | 0.43 |
| 121 | AGG325587 | EA | 100 | 49 | 1.12 | 0.11 | 0.59 | 0.05 | 1.71 | 0.17 |
| 122 | AGG325588 | EA | 100 | 53 | 1.07 | 0.36 | 0.29 | 0.21 | 1.36 | 0.58 |
| 123 | AGG325589 | SEA | 95 | 70 | 1.20 | 0.18 | 0.29 | 0.06 | 1.49 | 0.25 |
| 124 | AGG325590 | SWA | 100 | 71 | 0.72 | 0.19 | 0.27 | 0.05 | 0.98 | 0.25 |
| 125 | AGG325591 | SEA | 98 | 21 | 0.52 | 0.08 | 0.23 | 0.03 | 0.76 | 0.12 |
| 126 | AGG325593 | SA | 98 | 57 | 0.70 | 0.34 | 0.28 | 0.11 | 0.98 | 0.46 |
| 127 | AGG325594 | SWA | 92 | 33 | 0.99 | 0.09 | 0.24 | 0.04 | 1.23 | 0.13 |
| 128 | AGG325595 | AFR | 100 | 53 | 0.89 | 0.28 | 0.20 | 0.11 | 1.09 | 0.41 |
| 129 | AGG325596 | OP | 98 | 32 | 1.18 | 0.16 | 0.51 | 0.38 | 1.69 | 0.55 |
| 130 | AGG325597 | SEA | 100 | 47 | 1.14 | 0.15 | 0.46 | 0.10 | 1.60 | 0.26 |
| 131 | AGG325598 | SEA | 98 | 67 | 1.12 | 0.65 | 0.39 | 0.30 | 1.50 | 0.96 |
| 132 | AGG325599 | SEA | 100 | 17 | 1.11 | 0.11 | 0.24 | 0.04 | 1.36 | 0.16 |
| 133 | AGG325600 | SEA | 100 | 63 | 1.44 | 0.27 | 0.46 | 0.09 | 1.90 | 0.37 |
| 134 | AGG325601 | SWA | 100 | 49 | 0.51 | 0.37 | 0.17 | 0.12 | 0.69 | 0.05 |
| 135 | AGG325602 | SWA | 100 | 19 | 1.43 | 0.08 | 0.81 | 0.04 | 2.23 | 0.13 |
| 136 | AGG325603 | SWA | 100 | 46 | 0.76 | 0.16 | 0.32 | 0.07 | 1.08 | 0.24 |
| 137 | AGG325604 | SWA | 100 | 43 | 1.32 | 0.15 | 0.28 | 0.09 | 1.59 | 0.25 |
| 138 | AGG325605 | SWA | 100 | 35 | 0.89 | 0.17 | 0.39 | 0.08 | 1.28 | 0.26 |
| 139 | AGG325606 | SWA | 100 | 30 | 0.46 | 0.06 | 0.13 | 0.02 | 0.59 | 0.09 |
| 140 | AGG325607 | SWA | 94 | 14 | 1.13 | 0.05 | 0.35 | 0.02 | 1.49 | 0.08 |
| 141 | AGG325608 | SA | 100 | 64 | 0.52 | 0.14 | 0.20 | 0.08 | 0.72 | 0.22 |
| 142 | AGG325609 | SA | 100 | 33 | 0.88 | 0.05 | 0.21 | 0.03 | 1.09 | 0.09 |
| 143 | AGG325610 | SA | 95 | 28 | 0.29 | 0.09 | 0.15 | 0.03 | 0.44 | 0.14 |
| 144 | AGG325611 | SA | 95 | 15 | 0.35 | 0.04 | 0.29 | 0.04 | 0.65 | 0.09 |
| 145 | AGG325612 | SA | 96 | 96 | 0.42 | 0.27 | 0.10 | 0.08 | 0.52 | 0.36 |
| 146 | AGG325613 | UK | 95 | 28 | 0.63 | 0.18 | 0.26 | 0.09 | 0.89 | 0.28 |
| 147 | AGG325614 | UK | 92 | 21 | 0.62 | 0.04 | 0.25 | 0.03 | 0.86 | 0.08 |
| 148 | AGG325615 | SA | 94 | 23 | 0.79 | 0.01 | 0.17 | 0.01 | 0.96 | 0.03 |
| 149 | AGG325616 | SA | 96 | 28 | 0.70 | 0.04 | 0.22 | 0.02 | 0.92 | 0.05 |
| 150 | AGG325617 | SA | 100 | 60 | 0.33 | 0.29 | 0.39 | 0.10 | 0.72 | 0.40 |
| 151 | AGG325618 | SA | 100 | 45 | 0.94 | 0.15 | 0.22 | 0.07 | 1.17 | 0.23 |
| 152 | AGG325619 | SA | 100 | 51 | 0.69 | 0.11 | 0.15 | 0.04 | 0.85 | 0.13 |
| 153 | AGG325620 | SA | 95 | 66 | 0.79 | 0.28 | 0.17 | 0.11 | 0.96 | 0.37 |
| 154 | AGG325621 | SA | 95 | 70 | 1.33 | 0.37 | 0.36 | 0.13 | 1.69 | 0.48 |
| 155 | AGG325622 | SA | 100 | 72 | 1.17 | 0.31 | 0.22 | 0.14 | 1.38 | 0.43 |
| 156 | AGG325623 | SA | 97 | 42 | 0.66 | 0.13 | 0.19 | 0.07 | 0.85 | 0.18 |
| 157 | AGG325624 | SA | 100 | 42 | 0.71 | 0.20 | 0.22 | 0.07 | 0.93 | 0.26 |
| 158 | AGG325625 | SA | 100 | 55 | 0.75 | 0.10 | 0.21 | 0.02 | 0.96 | 0.13 |
| 159 | AGG325626 | SA | 100 | 66 | 1.20 | 0.61 | 0.32 | 0.17 | 1.52 | 0.78 |
| 160 | AGG325627 | SA | 100 | 51 | 0.94 | 0.55 | 0.28 | 0.18 | 1.22 | 0.74 |
| 161 | AGG325628 | SA | 97 | 25 | 1.43 | 0.29 | 0.30 | 0.14 | 1.73 | 0.41 |
| 162 | AGG325629 | SA | 100 | 40 | 1.01 | 0.12 | 0.20 | 0.03 | 1.21 | 0.13 |
| 163 | AGG325630 | SA | 100 | 74 | 1.36 | 0.41 | 0.32 | 0.17 | 1.68 | 0.57 |
| 164 | AGG325631 | SA | 98 | 79 | 0.63 | 0.47 | 0.15 | 0.11 | 0.78 | 0.57 |
| 165 | AGG325632 | SA | 100 | 60 | 1.15 | 0.19 | 0.31 | 0.15 | 1.46 | 0.33 |
| 166 | AGG325633 | SA | 100 | 44 | 1.39 | 0.28 | 0.41 | 0.08 | 1.81 | 0.35 |
| 167 | AGG325634 | SA | 100 | 54 | 0.96 | 0.24 | 0.26 | 0.07 | 1.23 | 0.30 |
| 168 | AGG325635 | SA | 97 | 57 | 0.71 | 0.49 | 0.29 | 0.20 | 1.00 | 0.70 |
| 169 | AGG325636 | SA | 97 | 75 | 0.22 | 0.14 | 0.07 | 0.04 | 0.29 | 0.18 |
| 170 | AGG325637 | SA | 97 | 57 | 0.58 | 0.37 | 0.21 | 0.13 | 0.80 | 0.49 |
| 171 | AGG325638 | SA | 97 | 55 | 0.58 | 0.29 | 0.17 | 0.11 | 0.75 | 0.38 |
| 172 | AGG325639 | SA | 100 | 37 | 1.11 | 0.04 | 0.24 | 0.18 | 1.34 | 0.22 |
| 173 | AGG325640 | SA | 96 | 78 | 0.76 | 0.37 | 0.26 | 0.17 | 1.02 | 0.53 |
| 174 | AGG325641 | SA | 92 | 10 | 0.87 | 0.05 | 0.24 | 0.02 | 1.11 | 0.07 |
| 175 | AGG325642 | SA | 100 | 70 | 1.49 | 0.35 | 0.32 | 0.11 | 1.81 | 0.45 |
| 176 | AGG325643 | SA | 96 | 76 | 0.60 | 0.44 | 0.31 | 0.24 | 0.91 | 0.65 |
| 177 | AGG325644 | SA | 92 | 62 | 0.84 | 0.36 | 0.24 | 0.06 | 1.07 | 0.42 |
| 178 | AGG325645 | SA | 96 | 80 | 0.48 | 0.09 | 0.16 | 0.03 | 0.63 | 0.11 |
| 179 | AGG325646 | SA | 100 | 15 | 0.70 | 0.08 | 0.19 | 0.02 | 0.88 | 0.09 |
| 180 | AGG325647 | SA | 95 | 54 | 0.75 | 0.58 | 0.23 | 0.19 | 0.98 | 0.77 |
| 181 | AGG325648 | SA | 96 | 40 | 0.42 | 0.13 | 0.11 | 0.05 | 0.52 | 0.17 |
| 182 | AGG325649 | UK | 100 | 55 | 1.08 | 0.19 | 0.27 | 0.07 | 1.35 | 0.24 |
| 183 | AGG325650 | SA | 97 | 78 | 1.01 | 0.27 | 0.23 | 0.11 | 1.24 | 0.36 |
| 184 | AGG325651 | SA | 97 | 9 | 1.18 | 0.11 | 0.29 | 0.04 | 1.46 | 0.15 |
| 185 | AGG325652 | SA | 97 | 46 | 0.45 | 0.07 | 0.20 | 0.03 | 0.65 | 0.08 |
| 186 | AGG325653 | SA | 100 | 86 | 0.53 | 0.10 | 0.23 | 0.03 | 0.76 | 0.12 |
| 187 | AGG325654 | SA | 97 | 71 | 0.70 | 0.13 | 0.31 | 0.06 | 1.01 | 0.19 |
| 188 | AGG325655 | SA | 98 | 39 | 0.25 | 0.03 | 0.08 | 0.01 | 0.33 | 0.02 |
| 189 | AGG325656 | SA | 97 | 40 | 0.89 | 0.17 | 0.31 | 0.08 | 1.20 | 0.24 |
| 190 | AGG325657 | SA | 100 | 27 | 1.05 | 0.10 | 0.31 | 0.05 | 1.36 | 0.15 |
| 191 | AGG325658 | SA | 98 | 49 | 0.74 | 0.32 | 0.21 | 0.05 | 0.94 | 0.36 |
| 192 | AGG325659 | SA | 93 | 35 | 0.82 | 0.12 | 0.24 | 0.02 | 1.06 | 0.12 |
| 193 | AGG325660 | SA | 97 | 74 | 0.57 | 0.20 | 0.20 | 0.09 | 0.78 | 0.28 |
| 194 | AGG325662 | SA | 100 | 51 | 1.05 | 0.16 | 0.32 | 0.12 | 1.37 | 0.28 |
| 195 | AGG325663 | SA | 97 | 60 | 0.44 | 0.07 | 0.13 | 0.06 | 0.57 | 0.13 |
| 196 | AGG325664 | SA | 96 | 32 | 0.93 | 0.10 | 0.27 | 0.16 | 1.20 | 0.36 |
| 197 | AGG325665 | SA | 98 | 61 | 1.33 | 0.29 | 0.40 | 0.10 | 1.73 | 0.41 |
| 198 | AGG325666 | SA | 95 | 57 | 1.17 | 0.15 | 0.27 | 0.04 | 1.44 | 0.16 |
| 199 | AGG325667 | SA | 100 | 92 | 0.93 | 0.35 | 0.27 | 0.10 | 1.20 | 0.44 |
| 200 | AGG325668 | SA | 100 | 66 | 0.97 | 0.59 | 0.29 | 0.20 | 1.26 | 0.78 |
| 201 | AGG325669 | SA | 100 | 62 | 0.96 | 0.21 | 0.32 | 0.04 | 1.28 | 0.23 |
| 202 | AGG325670 | SA | 100 | 80 | 1.33 | 0.38 | 0.40 | 0.13 | 1.73 | 0.49 |
| 203 | AGG325671 | SA | 97 | 85 | 0.12 | 0.05 | 0.15 | 0.02 | 0.27 | 0.07 |
| 204 | AGG325672 | SA | 100 | 85 | 0.82 | 0.14 | 0.21 | 0.06 | 1.03 | 0.18 |
| 205 | AGG325673 | SA | 92 | 47 | 1.17 | 0.27 | 0.47 | 0.14 | 1.64 | 0.41 |
| 206 | AGG325674 | SA | 100 | 61 | 0.73 | 0.05 | 0.19 | 0.02 | 0.92 | 0.05 |
| 207 | AGG325675 | SA | 100 | 52 | 1.14 | 0.26 | 0.28 | 0.08 | 1.41 | 0.33 |
| 208 | AGG325676 | SA | 100 | 55 | 1.03 | 0.34 | 0.28 | 0.09 | 1.31 | 0.42 |
| 209 | AGG325677 | SA | 100 | 13 | 1.16 | 0.10 | 0.30 | 0.03 | 1.47 | 0.13 |
| 210 | AGG325678 | SA | 100 | 62 | 1.08 | 0.30 | 0.27 | 0.15 | 1.35 | 0.44 |
| 211 | AGG325679 | SA | 98 | 42 | 0.33 | 0.17 | 0.11 | 0.08 | 0.44 | 0.24 |
| 212 | AGG325680 | SA | 96 | 80 | 0.70 | 0.35 | 0.27 | 0.08 | 0.97 | 0.42 |
| 213 | AGG325681 | SA | 95 | 37 | 1.39 | 0.20 | 0.33 | 0.09 | 1.72 | 0.27 |
| 214 | AGG325682 | SA | 95 | 44 | 1.44 | 0.38 | 0.35 | 0.18 | 1.79 | 0.54 |
| 215 | AGG325683 | SWA | 99 | 15 | 0.80 | 0.10 | 0.26 | 0.06 | 1.06 | 0.16 |
| 216 | AGG325684 | SA | 100 | 70 | 1.03 | 0.38 | 0.31 | 0.15 | 1.34 | 0.67 |
| 217 | AGG325685 | SA | 100 | 55 | 1.68 | 0.30 | 0.37 | 0.12 | 2.05 | 0.40 |
| 218 | AGG325686 | SA | 100 | 70 | 1.09 | 0.51 | 0.30 | 0.22 | 1.39 | 0.72 |
| 219 | AGG325687 | SA | 92 | 35 | 0.98 | 0.28 | 0.22 | 0.11 | 1.20 | 0.39 |
| 220 | AGG325688 | SWA | 95 | 50 | 0.97 | 0.41 | 0.31 | 0.13 | 1.28 | 0.54 |
| 221 | AGG325689 | SA | 95 | 44 | 0.95 | 0.26 | 0.26 | 0.11 | 1.21 | 0.35 |
| 222 | AGG325690 | SA | 92 | 20 | 0.53 | 0.07 | 0.25 | 0.03 | 0.77 | 0.08 |
| 223 | AGG325691 | SA | 100 | 60 | 0.57 | 0.19 | 0.13 | 0.06 | 0.70 | 0.24 |
| 224 | AGG325692 | SA | 92 | 32 | 0.42 | 0.14 | 0.20 | 0.14 | 0.62 | 0.27 |
| 225 | AGG325693 | SWA | 98 | 70 | 0.92 | 0.42 | 0.27 | 0.17 | 1.18 | 0.58 |
| 226 | AGG325694 | SWA | 95 | 10 | 0.73 | 0.11 | 0.21 | 0.04 | 0.95 | 0.13 |
| 227 | AGG325695 | SA | 95 | 64 | 0.93 | 0.23 | 0.23 | 0.09 | 1.15 | 0.31 |
| 228 | AGG325696 | SA | 93 | 5 | 0.60 | 0.06 | 0.27 | 0.02 | 0.87 | 0.09 |
| 229 | AGG325697 | SA | 97 | 58 | 1.12 | 0.55 | 0.31 | 0.17 | 1.43 | 0.71 |
| 230 | AGG325698 | SA | 97 | 12 | 1.05 | 0.09 | 0.33 | 0.02 | 1.39 | 0.09 |
| 231 | AGG325699 | SA | 99 | 77 | 1.02 | 0.57 | 0.30 | 0.18 | 1.33 | 0.76 |
| 232 | AGG325700 | SA | 100 | 19 | 0.84 | 0.04 | 0.23 | 0.01 | 1.07 | 0.03 |
| 233 | AGG325701 | SA | 92 | 76 | 0.77 | 0.51 | 0.27 | 0.25 | 1.04 | 0.96 |
| 234 | AGG325702 | SA | 92 | 34 | 1.07 | 0.18 | 0.24 | 0.10 | 1.31 | 0.27 |
| 235 | AGG325703 | SA | 95 | 84 | 1.09 | 0.58 | 0.25 | 0.16 | 1.34 | 0.74 |
| 236 | AGG325704 | OP | 97 | 67 | 1.57 | 0.23 | 0.30 | 0.07 | 1.88 | 0.28 |
| 237 | AGG325705 | SA | 96 | 61 | 0.73 | 0.23 | 0.22 | 0.07 | 0.95 | 0.28 |
| 238 | AGG325706 | SA | 94 | 20 | 1.30 | 0.10 | 0.32 | 0.05 | 1.63 | 0.16 |
| 239 | AGG325707 | SA | 94 | 63 | 0.88 | 0.69 | 0.20 | 0.19 | 1.08 | 0.86 |
| 240 | AGG325708 | SA | 94 | 50 | 0.94 | 0.12 | 0.27 | 0.06 | 1.22 | 0.16 |
| 241 | AGG325709 | SA | 97 | 45 | 1.02 | 0.08 | 0.22 | 0.02 | 1.24 | 0.08 |
| 242 | AGG325710 | SA | 97 | 62 | 0.51 | 0.09 | 0.10 | 0.05 | 0.61 | 0.13 |
| 243 | AGG325711 | UK | 96 | 70 | 0.63 | 0.45 | 0.27 | 0.19 | 0.90 | 0.62 |
| 244 | AGG325712 | SA | 97 | 32 | 1.15 | 0.18 | 0.28 | 0.06 | 1.42 | 0.24 |
| 245 | AGG325713 | SWA | 99 | 50 | 0.52 | 0.17 | 0.15 | 0.07 | 0.67 | 0.23 |
| 246 | AGG325714 | EUR | 98 | 32 | 0.90 | 0.20 | 0.22 | 0.08 | 1.13 | 0.26 |
| 247 | AGG325715 | SWA | 97 | 30 | 0.76 | 0.15 | 0.20 | 0.07 | 0.96 | 0.20 |
| 248 | AGG325716 | SA | 98 | 55 | 0.83 | 0.26 | 0.23 | 0.13 | 1.07 | 0.38 |
| 249 | AGG325717 | SWA | 93 | 55 | 0.93 | 0.55 | 0.32 | 0.17 | 1.25 | 0.73 |
| 250 | AGG325718 | SWA | 95 | 65 | 0.88 | 0.42 | 0.28 | 0.20 | 1.16 | 0.62 |
| 251 | AGG325719 | SWA | 94 | 45 | 0.82 | 0.26 | 0.28 | 0.13 | 1.10 | 0.40 |
| 252 | AGG325720 | SA | 97 | 25 | 1.12 | 0.06 | 0.30 | 0.02 | 1.42 | 0.07 |
| 253 | AGG325721 | SA | 99 | 37 | 1.35 | 0.19 | 0.36 | 0.06 | 1.71 | 0.25 |
| 254 | AGG325722 | SA | 98 | 70 | 0.72 | 0.18 | 0.23 | 0.09 | 0.94 | 0.26 |
| 255 | AGG325723 | SWA | 94 | 37 | 0.83 | 0.25 | 0.24 | 0.08 | 1.06 | 0.33 |
| 256 | AGG325724 | SWA | 95 | 42 | 1.10 | 0.32 | 0.34 | 0.15 | 1.44 | 0.47 |
| 257 | AGG325725 | SWA | 96 | 30 | 1.33 | 0.12 | 0.35 | 0.07 | 1.69 | 0.17 |
| 258 | AGG325726 | SWA | 92 | 35 | 0.30 | 0.21 | 0.06 | 0.04 | 0.36 | 0.24 |
| 259 | AGG325727 | SWA | 94 | 40 | 1.07 | 0.14 | 0.29 | 0.07 | 1.37 | 0.22 |
| 260 | AGG325728 | SWA | 95 | 68 | 1.03 | 0.33 | 0.33 | 0.13 | 1.36 | 0.45 |
| 261 | AGG325729 | SWA | 96 | 65 | 1.12 | 0.36 | 0.39 | 0.15 | 1.51 | 0.51 |
| 262 | AGG325730 | SWA | 98 | 50 | 1.12 | 0.43 | 0.29 | 0.17 | 1.41 | 0.59 |
| 263 | AGG325731 | SWA | 93 | 22 | 1.32 | 0.21 | 0.29 | 0.10 | 1.61 | 0.31 |
| 264 | AGG325732 | SA | 95 | 78 | 0.43 | 0.07 | 0.16 | 0.02 | 0.58 | 0.07 |
| 265 | AGG325733 | SA | 95 | 59 | 1.42 | 0.41 | 0.40 | 0.18 | 1.82 | 0.58 |
| 266 | AGG325734 | SA | 97 | 47 | 1.03 | 0.43 | 0.26 | 0.09 | 1.29 | 0.50 |
| 267 | AGG325735 | SA | 98 | 62 | 0.69 | 0.16 | 0.23 | 0.04 | 0.91 | 0.20 |
| 268 | AGG325736 | SA | 97 | 47 | 0.82 | 0.22 | 0.27 | 0.08 | 1.09 | 0.29 |
| 269 | AGG325737 | SA | 98 | 52 | 0.85 | 0.12 | 0.25 | 0.05 | 1.10 | 0.16 |
| 270 | AGG325738 | SA | 97 | 67 | 0.82 | 0.36 | 0.21 | 0.09 | 1.03 | 0.43 |
| 271 | AGG325739 | SA | 98 | 52 | 0.70 | 0.25 | 0.18 | 0.08 | 0.88 | 0.32 |
| 272 | AGG325740 | SA | 96 | 64 | 0.91 | 0.43 | 0.22 | 0.19 | 1.13 | 0.62 |
| 273 | AGG325741 | SA | 96 | 69 | 0.68 | 0.27 | 0.21 | 0.08 | 0.89 | 0.34 |
| 274 | AGG325742 | SA | 98 | 80 | 1.54 | 0.24 | 0.27 | 0.07 | 1.81 | 0.31 |
| 275 | AGG325743 | SWA | 96 | 30 | 0.96 | 0.28 | 0.27 | 0.10 | 1.23 | 0.38 |
| 276 | AGG325744 | SWA | 96 | 56 | 0.82 | 0.21 | 0.21 | 0.07 | 1.03 | 0.28 |
| 277 | AGG325745 | SWA | 95 | 75 | 0.72 | 0.32 | 0.24 | 0.16 | 0.95 | 0.47 |
| 278 | AGG325746 | SWA | 97 | 60 | 1.05 | 0.14 | 0.24 | 0.18 | 1.28 | 0.31 |
| 279 | AGG325747 | SWA | 95 | 42 | 1.15 | 0.19 | 0.27 | 0.09 | 1.42 | 0.28 |
| 280 | AGG325748 | SWA | 97 | 45 | 0.86 | 0.04 | 0.35 | 0.02 | 1.21 | 0.05 |
| 281 | AGG325749 | SWA | 93 | 5 | 0.95 | 0.03 | 0.22 | 0.01 | 1.18 | 0.02 |
| 282 | AGG325750 | SWA | 94 | 75 | 0.98 | 0.35 | 0.24 | 0.14 | 1.22 | 0.48 |
| 283 | AGG325751 | SWA | 95 | 63 | 1.26 | 0.42 | 0.31 | 0.14 | 1.57 | 0.56 |
| 284 | AGG325752 | SWA | 98 | 62 | 1.22 | 0.50 | 0.32 | 0.16 | 1.53 | 0.65 |
| 285 | AGG325753 | SWA | 97 | 79 | 0.55 | 0.37 | 0.19 | 0.14 | 0.75 | 0.49 |
| 286 | AGG325754 | SWA | 98 | 74 | 1.11 | 0.29 | 0.30 | 0.11 | 1.41 | 0.38 |
| 287 | AGG325755 | SA | 97 | 19 | 0.41 | 0.04 | 0.18 | 0.02 | 0.59 | 0.06 |
| 288 | AGG325756 | SA | 96 | 15 | 0.87 | 0.04 | 0.25 | 0.01 | 1.12 | 0.03 |
| 289 | AGG325758 | MA | 94 | 42 | 0.86 | 0.23 | 0.21 | 0.10 | 1.06 | 0.32 |
| 290 | AGG325759 | UK | 96 | 50 | 1.23 | 0.35 | 0.40 | 0.12 | 1.64 | 0.46 |
| 291 | AGG325760 | SA | 98 | 86 | 1.69 | 0.79 | 0.57 | 0.30 | 2.26 | 1.08 |
| 292 | AGG325761 | AFR | 96 | 17 | 0.99 | 0.10 | 0.21 | 0.04 | 1.20 | 0.12 |

Supplementary Table 13| Genotype means from screening of 292 mungbean mini−core genotypes for waterlogging tolerance at the seedling stage. The treatment was waterlogged for 8 days at the seedling stage and compared with their respective drained control. The waterlogged pots recovered for 7 days immediately after the release of waterlogging. Harvesting of waterlogged and control pots occurred on the same day. Data represent the mean value for three replications.

| **Sr. No.** | **Accession Number** | **Origin** | **Seedling stage** | | | | | | | | | |
| --- | --- | --- | --- | --- | --- | --- | --- | --- | --- | --- | --- | --- |
|  |  |  | **Shoot dry mass (g)** | | **Root dry mass (g)** | | **Total dry mass (g)** | | **No. of adventitious roots** | **SPAD chlorophyll content** | | **100- seed weight (g)** |
|  |  |  | **Control** | **WL** | **Control** | **WL** | **Control** | **WL** |  | **Control** | **WL** |  |
| 1 | AGG325466 | SEA | 1.44 | 0.73 | 0.41 | 0.28 | 1.85 | 1.01 | 17 | 37 | 26 | 6.7 |
| 2 | AGG325467 | SA | 0.44 | 0.32 | 0.25 | 0.11 | 0.69 | 0.43 | 12 | 40 | 34 | 3.2 |
| 3 | AGG325468 | SA | 0.71 | 0.42 | 0.39 | 0.15 | 1.10 | 0.57 | 14 | 41 | 33 | 3.1 |
| 4 | AGG325469 | SWA | 0.68 | 0.52 | 0.35 | 0.18 | 1.03 | 0.70 | 12 | 38 | 32 | 5.3 |
| 5 | AGG325470 | SWA | 0.40 | 0.23 | 0.13 | 0.07 | 0.52 | 0.31 | 6 | 40 | 31 | 3.1 |
| 6 | AGG325471 | SA | 0.81 | 0.38 | 0.29 | 0.15 | 1.10 | 0.53 | 10 | 35 | 22 | 2.9 |
| 7 | AGG325472 | SWA | 0.80 | 0.66 | 0.30 | 0.21 | 1.10 | 0.87 | 12 | 35 | 32 | 3.3 |
| 8 | AGG325473 | SWA | 0.51 | 0.36 | 0.25 | 0.12 | 0.76 | 0.48 | 10 | 46 | 34 | 3.7 |
| 9 | AGG325474 | NA | 0.56 | 0.30 | 0.23 | 0.06 | 0.79 | 0.36 | 3 | 39 | 31 | 3.3 |
| 10 | AGG325475 | SWA | 0.72 | 0.45 | 0.34 | 0.14 | 1.06 | 0.59 | 11 | 39 | 30 | 2.6 |
| 11 | AGG325476 | SWA | 0.39 | 0.24 | 0.22 | 0.08 | 0.61 | 0.32 | 6 | 40 | 30 | 2.9 |
| 12 | AGG325477 | SA | 0.89 | 0.41 | 0.43 | 0.14 | 1.31 | 0.55 | 15 | 38 | 32 | 3.0 |
| 13 | AGG325478 | SWA | 0.88 | 0.37 | 0.26 | 0.10 | 1.14 | 0.46 | 6 | 40 | 29 | 3.6 |
| 14 | AGG325479 | SWA | 1.16 | 0.30 | 0.28 | 0.02 | 1.44 | 0.32 | 0 | 43 | 29 | 2.9 |
| 15 | AGG325480 | SWA | 0.94 | 0.51 | 0.32 | 0.12 | 1.27 | 0.63 | 12 | 42 | 34 | 3.8 |
| 16 | AGG325481 | SEA | 1.52 | 0.96 | 0.57 | 0.31 | 2.08 | 1.27 | 16 | 39 | 30 | 3.7 |
| 17 | AGG325482 | SEA | 0.94 | 0.58 | 0.32 | 0.18 | 1.27 | 0.76 | 7 | 37 | 31 | 3.2 |
| 18 | AGG325483 | SWA | 1.60 | 0.85 | 0.50 | 0.24 | 2.10 | 1.08 | 15 | 41 | 27 | 4.2 |
| 19 | AGG325484 | SA | 0.54 | 0.14 | 0.25 | 0.05 | 0.79 | 0.19 | 3 | 40 | 29 | 3.3 |
| 20 | AGG325485 | SA | 0.65 | 0.41 | 0.20 | 0.11 | 0.85 | 0.51 | 14 | 38 | 31 | 3.6 |
| 21 | AGG325486 | SA | 0.56 | 0.25 | 0.31 | 0.08 | 0.87 | 0.33 | 4 | 39 | 30 | 3.7 |
| 22 | AGG325487 | SA | 0.57 | 0.31 | 0.24 | 0.07 | 0.81 | 0.38 | 4 | 31 | 28 | 3.2 |
| 23 | AGG325488 | SA | 0.60 | 0.19 | 0.18 | 0.09 | 0.78 | 0.28 | 3 | 38 | 30 | 3.5 |
| 24 | AGG325489 | SA | 0.42 | 0.27 | 0.23 | 0.12 | 0.65 | 0.39 | 12 | 41 | 31 | 3.8 |
| 25 | AGG325490 | SA | 0.67 | 0.45 | 0.41 | 0.13 | 1.08 | 0.58 | 16 | 36 | 30 | 4.1 |
| 26 | AGG325491 | SA | 1.13 | 0.47 | 0.45 | 0.15 | 1.58 | 0.62 | 18 | 38 | 31 | 3.2 |
| 27 | AGG325492 | SAM | 0.82 | 0.36 | 0.22 | 0.12 | 1.04 | 0.48 | 15 | 37 | 31 | 2.7 |
| 28 | AGG325493 | SA | 0.63 | 0.28 | 0.32 | 0.11 | 0.95 | 0.39 | 7 | 39 | 28 | 2.7 |
| 29 | AGG325494 | SA | 0.61 | 0.25 | 0.18 | 0.09 | 0.79 | 0.34 | 10 | 38 | 27 | 3.3 |
| 30 | AGG325495 | NA | 1.30 | 0.43 | 0.53 | 0.10 | 1.83 | 0.53 | 6 | 35 | 29 | 3.4 |
| 31 | AGG325496 | SWA | 0.55 | 0.32 | 0.32 | 0.10 | 0.87 | 0.42 | 10 | 43 | 38 | 3.3 |
| 32 | AGG325497 | SA | 0.66 | 0.46 | 0.21 | 0.10 | 0.87 | 0.57 | 14 | 39 | 32 | 3.5 |
| 33 | AGG325498 | SA | 1.11 | 0.39 | 0.37 | 0.10 | 1.48 | 0.48 | 8 | 36 | 26 | 2.6 |
| 34 | AGG325499 | SA | 0.95 | 0.46 | 0.26 | 0.09 | 1.21 | 0.54 | 9 | 42 | 31 | 3.4 |
| 35 | AGG325500 | SA | 0.59 | 0.38 | 0.27 | 0.14 | 0.86 | 0.52 | 11 | 41 | 32 | 3.8 |
| 36 | AGG325501 | SA | 0.67 | 0.24 | 0.25 | 0.07 | 0.92 | 0.31 | 16 | 39 | 30 | 5.2 |
| 37 | AGG325502 | SA | 0.86 | 0.49 | 0.36 | 0.13 | 1.21 | 0.62 | 10 | 39 | 31 | 3.4 |
| 38 | AGG325503 | SA | 1.23 | 0.60 | 0.36 | 0.16 | 1.59 | 0.77 | 13 | 41 | 25 | 3.3 |
| 39 | AGG325504 | SA | 0.65 | 0.35 | 0.24 | 0.10 | 0.89 | 0.45 | 6 | 43 | 27 | 4.3 |
| 40 | AGG325505 | SA | 0.75 | 0.46 | 0.30 | 0.17 | 1.04 | 0.63 | 15 | 40 | 27 | 5.7 |
| 41 | AGG325506 | SA | 1.07 | 0.74 | 0.47 | 0.23 | 1.53 | 0.97 | 18 | 43 | 32 | 3.6 |
| 42 | AGG325507 | SA | 0.66 | 0.22 | 0.20 | 0.06 | 0.86 | 0.28 | 6 | 40 | 27 | 3.4 |
| 43 | AGG325508 | SA | 1.53 | 0.69 | 0.35 | 0.21 | 1.87 | 0.90 | 16 | 41 | 34 | 2.7 |
| 44 | AGG325509 | SA | 1.27 | 0.51 | 0.32 | 0.16 | 1.59 | 0.67 | 8 | 44 | 29 | 3.5 |
| 45 | AGG325510 | SEA | 1.08 | 0.38 | 0.51 | 0.17 | 1.59 | 0.55 | 14 | 37 | 29 | 3.0 |
| 46 | AGG325511 | SA | 0.88 | 0.41 | 0.34 | 0.15 | 1.23 | 0.56 | 7 | 38 | 24 | 3.2 |
| 47 | AGG325512 | OP | 0.80 | 0.32 | 0.43 | 0.21 | 1.24 | 0.53 | 14 | 45 | 31 | 3.2 |
| 48 | AGG325513 | OP | 1.28 | 0.62 | 0.36 | 0.21 | 1.64 | 0.83 | 15 | 41 | 36 | 3.4 |
| 49 | AGG325514 | OP | 1.28 | 0.96 | 0.52 | 0.32 | 1.80 | 1.28 | 22 | 47 | 37 | 2.9 |
| 50 | AGG325515 | OP | 0.73 | 0.34 | 0.25 | 0.13 | 0.97 | 0.47 | 11 | 37 | 28 | 3.1 |
| 51 | AGG325516 | OP | 0.49 | 0.25 | 0.20 | 0.11 | 0.69 | 0.36 | 11 | 42 | 32 | 3.0 |
| 52 | AGG325517 | SEA | 0.87 | 0.55 | 0.35 | 0.17 | 1.22 | 0.72 | 16 | 37 | 28 | 3.7 |
| 53 | AGG325518 | SEA | 1.03 | 0.18 | 0.32 | 0.07 | 1.35 | 0.25 | 11 | 39 | 27 | 3.2 |
| 54 | AGG325519 | SEA | 0.99 | 0.51 | 0.42 | 0.14 | 1.42 | 0.65 | 9 | 41 | 28 | 6.1 |
| 55 | AGG325520 | SEA | 1.32 | 0.65 | 0.44 | 0.17 | 1.76 | 0.83 | 15 | 43 | 29 | 4.1 |
| 56 | AGG325521 | SA | 0.86 | 0.40 | 0.28 | 0.07 | 1.14 | 0.47 | 11 | 38 | 31 | 2.9 |
| 57 | AGG325522 | SA | 0.54 | 0.28 | 0.23 | 0.08 | 0.77 | 0.36 | 7 | 35 | 25 | 3.1 |
| 58 | AGG325523 | SA | 0.76 | 0.36 | 0.20 | 0.12 | 0.96 | 0.48 | 13 | 40 | 30 | 5.1 |
| 59 | AGG325524 | SEA | 0.80 | 0.50 | 0.39 | 0.18 | 1.20 | 0.68 | 12 | 39 | 33 | 3.4 |
| 60 | AGG325525 | SA | 0.46 | 0.22 | 0.21 | 0.10 | 0.67 | 0.32 | 8 | 36 | 25 | 3.4 |
| 61 | AGG325526 | SA | 0.60 | 0.36 | 0.22 | 0.11 | 0.82 | 0.46 | 11 | 36 | 30 | 3.6 |
| 62 | AGG325527 | SA | 0.86 | 0.29 | 0.35 | 0.11 | 1.21 | 0.39 | 9 | 44 | 34 | 3.1 |
| 63 | AGG325528 | SWA | 0.83 | 0.47 | 0.36 | 0.14 | 1.20 | 0.61 | 8 | 44 | 33 | 3.8 |
| 64 | AGG325529 | SA | 0.76 | 0.39 | 0.23 | 0.08 | 0.99 | 0.47 | 7 | 40 | 29 | 4.1 |
| 65 | AGG325530 | SA | 0.56 | 0.27 | 0.24 | 0.06 | 0.80 | 0.33 | 9 | 37 | 30 | 3.2 |
| 66 | AGG325531 | SA | 0.41 | 0.24 | 0.22 | 0.07 | 0.62 | 0.30 | 5 | 36 | 22 | 4.2 |
| 67 | AGG325532 | SA | 0.46 | 0.30 | 0.23 | 0.09 | 0.69 | 0.39 | 7 | 39 | 25 | 3.1 |
| 68 | AGG325533 | NA | 0.86 | 0.33 | 0.41 | 0.10 | 1.27 | 0.43 | 7 | 41 | 36 | 3.2 |
| 69 | AGG325534 | SA | 0.40 | 0.17 | 0.13 | 0.05 | 0.53 | 0.22 | 7 | 45 | 30 | 3.0 |
| 70 | AGG325535 | SA | 0.67 | 0.30 | 0.22 | 0.09 | 0.89 | 0.39 | 7 | 41 | 35 | 4.8 |
| 71 | AGG325536 | SA | 0.67 | 0.41 | 0.31 | 0.13 | 0.98 | 0.54 | 8 | 43 | 36 | 3.1 |
| 72 | AGG325537 | SWA | 0.94 | 0.55 | 0.29 | 0.14 | 1.23 | 0.69 | 12 | 42 | 35 | 3.2 |
| 73 | AGG325538 | SWA | 0.96 | 0.46 | 0.41 | 0.16 | 1.37 | 0.62 | 10 | 37 | 35 | 3.3 |
| 74 | AGG325539 | SA | 0.57 | 0.28 | 0.27 | 0.09 | 0.83 | 0.36 | 9 | 42 | 30 | 3.2 |
| 75 | AGG325540 | SA | 0.58 | 0.23 | 0.35 | 0.06 | 0.93 | 0.29 | 9 | 38 | 24 | 3.2 |
| 76 | AGG325541 | SA | 0.42 | 0.21 | 0.16 | 0.07 | 0.57 | 0.28 | 10 | 37 | 24 | 3.0 |
| 77 | AGG325542 | SA | 0.69 | 0.47 | 0.35 | 0.17 | 1.04 | 0.64 | 18 | 42 | 28 | 3.7 |
| 78 | AGG325543 | SA | 0.76 | 0.31 | 0.20 | 0.10 | 0.96 | 0.42 | 12 | 38 | 31 | 3.0 |
| 79 | AGG325544 | SA | 0.41 | 0.17 | 0.19 | 0.06 | 0.60 | 0.23 | 10 | 40 | 30 | 3.5 |
| 80 | AGG325545 | SA | 0.51 | 0.35 | 0.25 | 0.08 | 0.77 | 0.43 | 7 | 43 | 30 | 3.4 |
| 81 | AGG325546 | NA | 0.63 | 0.48 | 0.21 | 0.12 | 0.84 | 0.60 | 12 | 40 | 26 | 3.6 |
| 82 | AGG325547 | SA | 0.77 | 0.36 | 0.22 | 0.12 | 0.99 | 0.48 | 16 | 37 | 33 | 5.8 |
| 83 | AGG325548 | SA | 0.58 | 0.53 | 0.25 | 0.14 | 0.82 | 0.67 | 13 | 34 | 30 | 3.4 |
| 84 | AGG325549 | SA | 1.05 | 0.39 | 0.44 | 0.14 | 1.49 | 0.53 | 10 | 39 | 29 | 3.3 |
| 85 | AGG325550 | SA | 1.07 | 0.46 | 0.35 | 0.13 | 1.43 | 0.59 | 10 | 39 | 30 | 2.6 |
| 86 | AGG325551 | UK | 0.81 | 0.33 | 0.34 | 0.11 | 1.14 | 0.44 | 19 | 38 | 29 | 3.7 |
| 87 | AGG325552 | SA | 0.64 | 0.30 | 0.20 | 0.08 | 0.84 | 0.38 | 14 | 41 | 29 | 3.3 |
| 88 | AGG325553 | SA | 0.64 | 0.34 | 0.39 | 0.10 | 1.03 | 0.44 | 13 | 36 | 31 | 3.0 |
| 89 | AGG325554 | SA | 0.72 | 0.46 | 0.26 | 0.13 | 0.98 | 0.59 | 13 | 43 | 35 | 3.7 |
| 90 | AGG325555 | SA | 0.76 | 0.46 | 0.29 | 0.15 | 1.05 | 0.61 | 15 | 40 | 32 | 5.1 |
| 91 | AGG325556 | SA | 1.11 | 0.57 | 0.35 | 0.19 | 1.46 | 0.76 | 17 | 36 | 26 | 3.8 |
| 92 | AGG325557 | SA | 0.58 | 0.23 | 0.23 | 0.09 | 0.81 | 0.32 | 6 | 39 | 27 | 6.5 |
| 93 | AGG325558 | SA | 0.69 | 0.25 | 0.29 | 0.10 | 0.98 | 0.36 | 9 | 45 | 30 | 2.9 |
| 94 | AGG325559 | SA | 0.89 | 0.63 | 0.24 | 0.15 | 1.12 | 0.78 | 13 | 41 | 26 | 5.1 |
| 95 | AGG325560 | SA | 0.70 | 0.63 | 0.27 | 0.18 | 0.97 | 0.81 | 25 | 37 | 27 | 3.6 |
| 96 | AGG325561 | SA | 0.63 | 0.41 | 0.29 | 0.14 | 0.92 | 0.55 | 10 | 38 | 29 | 2.7 |
| 97 | AGG325562 | SA | 0.96 | 0.35 | 0.45 | 0.12 | 1.41 | 0.46 | 12 | 42 | 28 | 3.4 |
| 98 | AGG325563 | SA | 0.79 | 0.49 | 0.26 | 0.13 | 1.04 | 0.61 | 11 | 39 | 31 | 5.4 |
| 99 | AGG325564 | SWA | 0.55 | 0.38 | 0.28 | 0.16 | 0.83 | 0.54 | 14 | 36 | 27 | 3.8 |
| 100 | AGG325565 | SWA | 0.59 | 0.33 | 0.26 | 0.10 | 0.85 | 0.43 | 5 | 37 | 32 | 4.1 |
| 101 | AGG325566 | EUR | 0.73 | 0.33 | 0.32 | 0.11 | 1.05 | 0.44 | 8 | 38 | 29 | 3.0 |
| 102 | AGG325567 | SEA | 1.08 | 0.77 | 0.38 | 0.18 | 1.46 | 0.95 | 18 | 37 | 33 | 3.4 |
| 103 | AGG325568 | EA | 1.02 | 0.43 | 0.44 | 0.16 | 1.46 | 0.59 | 17 | 42 | 34 | 3.7 |
| 104 | AGG325569 | EA | 0.83 | 0.51 | 0.31 | 0.20 | 1.14 | 0.71 | 12 | 41 | 31 | 3.1 |
| 105 | AGG325570 | SA | 0.76 | 0.56 | 0.29 | 0.18 | 1.05 | 0.74 | 14 | 34 | 28 | 3.0 |
| 106 | AGG325571 | SA | 0.76 | 0.58 | 0.33 | 0.22 | 1.09 | 0.80 | 15 | 37 | 28 | 4.0 |
| 107 | AGG325572 | SA | 0.95 | 0.46 | 0.40 | 0.29 | 1.35 | 0.75 | 13 | 36 | 27 | 3.9 |
| 108 | AGG325573 | NA | 0.93 | 0.61 | 0.39 | 0.27 | 1.32 | 0.88 | 11 | 33 | 21 | 4.3 |
| 109 | AGG325574 | SA | 0.99 | 0.74 | 0.45 | 0.28 | 1.44 | 1.02 | 14 | 39 | 21 | 3.7 |
| 110 | AGG325575 | SA | 1.00 | 0.65 | 0.37 | 0.17 | 1.37 | 0.82 | 20 | 37 | 26 | 3.7 |
| 111 | AGG325576 | SA | 0.77 | 0.59 | 0.34 | 0.19 | 1.10 | 0.79 | 15 | 38 | 25 | 3.2 |
| 112 | AGG325577 | SA | 0.92 | 0.66 | 0.34 | 0.26 | 1.26 | 0.91 | 15 | 36 | 22 | 3.1 |
| 113 | AGG325578 | SA | 1.28 | 0.58 | 0.50 | 0.26 | 1.78 | 0.84 | 20 | 35 | 27 | 2.6 |
| 114 | AGG325579 | SEA | 1.09 | 0.63 | 0.51 | 0.30 | 1.61 | 0.93 | 18 | 40 | 22 | 3.1 |
| 115 | AGG325580 | EA | 1.03 | 0.63 | 0.39 | 0.21 | 1.42 | 0.84 | 13 | 38 | 23 | 2.6 |
| 116 | AGG325581 | SEA | 1.33 | 0.60 | 0.40 | 0.26 | 1.73 | 0.86 | 15 | 41 | 24 | 3.3 |
| 117 | AGG325582 | SWA | 0.98 | 0.35 | 0.36 | 0.20 | 1.34 | 0.56 | 12 | 37 | 27 | 3.0 |
| 118 | AGG325584 | SWA | 0.46 | 0.34 | 0.30 | 0.18 | 0.76 | 0.51 | 12 | 39 | 34 | 3.2 |
| 119 | AGG325585 | SEA | 0.91 | 0.43 | 0.33 | 0.18 | 1.25 | 0.61 | 12 | 37 | 25 | 4.6 |
| 120 | AGG325586 | SEA | 1.18 | 0.70 | 0.64 | 0.32 | 1.82 | 1.02 | 19 | 41 | 28 | 3.9 |
| 121 | AGG325587 | EA | 0.60 | 0.37 | 0.28 | 0.15 | 0.88 | 0.52 | 12 | 40 | 25 | 3.8 |
| 122 | AGG325588 | EA | 1.19 | 0.66 | 0.46 | 0.29 | 1.65 | 0.95 | 8 | 38 | 29 | 7.1 |
| 123 | AGG325589 | SEA | 1.02 | 0.85 | 0.46 | 0.33 | 1.48 | 1.18 | 15 | 38 | 26 | 3.5 |
| 124 | AGG325590 | SWA | 0.61 | 0.46 | 0.31 | 0.16 | 0.92 | 0.62 | 16 | 41 | 28 | 3.4 |
| 125 | AGG325591 | SEA | 1.26 | 0.84 | 0.48 | 0.36 | 1.74 | 1.21 | 20 | 34 | 27 | 3.8 |
| 126 | AGG325593 | SA | 1.09 | 0.66 | 0.68 | 0.23 | 1.77 | 0.89 | 23 | 43 | 30 | 6.0 |
| 127 | AGG325594 | SWA | 0.85 | 0.62 | 0.35 | 0.19 | 1.20 | 0.81 | 14 | 38 | 27 | 3.5 |
| 128 | AGG325595 | AFR | 0.93 | 0.60 | 0.38 | 0.23 | 1.30 | 0.82 | 16 | 37 | 27 | 4.6 |
| 129 | AGG325596 | OP | 1.39 | 0.84 | 0.53 | 0.27 | 1.92 | 1.11 | 18 | 41 | 30 | 3.2 |
| 130 | AGG325597 | SEA | 0.55 | 0.44 | 0.31 | 0.21 | 0.86 | 0.65 | 11 | 43 | 28 | 2.9 |
| 131 | AGG325598 | SEA | 0.99 | 0.58 | 0.62 | 0.22 | 1.61 | 0.80 | 16 | 37 | 28 | 4.9 |
| 132 | AGG325599 | SEA | 0.88 | 0.53 | 0.44 | 0.28 | 1.32 | 0.82 | 17 | 38 | 29 | 3.6 |
| 133 | AGG325600 | SEA | 0.83 | 0.66 | 0.46 | 0.29 | 1.28 | 0.95 | 20 | 36 | 28 | 6.4 |
| 134 | AGG325601 | SWA | 1.20 | 0.89 | 0.59 | 0.34 | 1.79 | 1.23 | 17 | 42 | 27 | 4.6 |
| 135 | AGG325602 | SWA | 1.37 | 0.65 | 0.65 | 0.27 | 2.02 | 0.92 | 14 | 39 | 30 | 2.4 |
| 136 | AGG325603 | SWA | 0.75 | 0.29 | 0.29 | 0.17 | 1.04 | 0.47 | 12 | 39 | 24 | 5.3 |
| 137 | AGG325604 | SWA | 0.66 | 0.35 | 0.46 | 0.18 | 1.12 | 0.53 | 8 | 41 | 26 | 7.1 |
| 138 | AGG325605 | SWA | 1.24 | 0.62 | 0.66 | 0.26 | 1.90 | 0.89 | 19 | 38 | 30 | 5.4 |
| 139 | AGG325606 | SWA | 1.06 | 0.68 | 0.38 | 0.22 | 1.45 | 0.90 | 13 | 36 | 27 | 3.9 |
| 140 | AGG325607 | SWA | 0.72 | 0.53 | 0.31 | 0.22 | 1.03 | 0.76 | 12 | 41 | 25 | 4.2 |
| 141 | AGG325608 | SA | 0.78 | 0.42 | 0.34 | 0.17 | 1.12 | 0.59 | 13 | 34 | 31 | 3.5 |
| 142 | AGG325609 | SA | 0.67 | 0.54 | 0.35 | 0.19 | 1.02 | 0.72 | 13 | 37 | 28 | 3.7 |
| 143 | AGG325610 | SA | 0.76 | 0.35 | 0.32 | 0.15 | 1.08 | 0.50 | 16 | 38 | 27 | 3.2 |
| 144 | AGG325611 | SA | 0.98 | 0.53 | 0.36 | 0.21 | 1.34 | 0.74 | 19 | 42 | 27 | 3.4 |
| 145 | AGG325612 | SA | 0.88 | 0.31 | 0.26 | 0.10 | 1.14 | 0.41 | 10 | 35 | 24 | 3.7 |
| 146 | AGG325613 | UK | 0.77 | 0.51 | 0.35 | 0.20 | 1.12 | 0.71 | 9 | 38 | 26 | 3.4 |
| 147 | AGG325614 | UK | 0.81 | 0.48 | 0.46 | 0.25 | 1.27 | 0.72 | 15 | 38 | 28 | 2.5 |
| 148 | AGG325615 | SA | 0.53 | 0.34 | 0.25 | 0.18 | 0.79 | 0.52 | 9 | 42 | 29 | 3.1 |
| 149 | AGG325616 | SA | 0.44 | 0.38 | 0.21 | 0.12 | 0.66 | 0.50 | 13 | 35 | 27 | 2.8 |
| 150 | AGG325617 | SA | 0.49 | 0.25 | 0.23 | 0.16 | 0.73 | 0.41 | 9 | 36 | 26 | 3.1 |
| 151 | AGG325618 | SA | 0.68 | 0.39 | 0.44 | 0.17 | 1.11 | 0.56 | 9 | 43 | 29 | 3.4 |
| 152 | AGG325619 | SA | 0.66 | 0.28 | 0.32 | 0.12 | 0.97 | 0.41 | 8 | 37 | 26 | 5.0 |
| 153 | AGG325620 | SA | 0.54 | 0.30 | 0.23 | 0.15 | 0.76 | 0.45 | 13 | 37 | 27 | 3.1 |
| 154 | AGG325621 | SA | 0.62 | 0.33 | 0.30 | 0.12 | 0.92 | 0.45 | 9 | 37 | 31 | 2.6 |
| 155 | AGG325622 | SA | 0.70 | 0.39 | 0.23 | 0.14 | 0.93 | 0.52 | 13 | 40 | 33 | 6.5 |
| 156 | AGG325623 | SA | 0.35 | 0.20 | 0.17 | 0.07 | 0.53 | 0.27 | 7 | 35 | 22 | 7.0 |
| 157 | AGG325624 | SA | 0.56 | 0.36 | 0.27 | 0.16 | 0.84 | 0.52 | 11 | 39 | 31 | 3.1 |
| 158 | AGG325625 | SA | 0.57 | 0.29 | 0.16 | 0.12 | 0.73 | 0.41 | 10 | 40 | 28 | 3.0 |
| 159 | AGG325626 | SA | 0.62 | 0.47 | 0.29 | 0.22 | 0.91 | 0.69 | 12 | 37 | 26 | 3.1 |
| 160 | AGG325627 | SA | 0.48 | 0.29 | 0.31 | 0.11 | 0.79 | 0.41 | 10 | 40 | 23 | 3.9 |
| 161 | AGG325628 | SA | 0.84 | 0.54 | 0.50 | 0.18 | 1.34 | 0.72 | 14 | 38 | 27 | 2.8 |
| 162 | AGG325629 | SA | 0.70 | 0.36 | 0.29 | 0.12 | 0.99 | 0.48 | 8 | 41 | 30 | 6.6 |
| 163 | AGG325630 | SA | 0.79 | 0.58 | 0.32 | 0.27 | 1.12 | 0.85 | 14 | 37 | 28 | 3.6 |
| 164 | AGG325631 | SA | 0.64 | 0.44 | 0.24 | 0.18 | 0.88 | 0.62 | 9 | 40 | 25 | 3.7 |
| 165 | AGG325632 | SA | 0.56 | 0.36 | 0.33 | 0.13 | 0.89 | 0.49 | 10 | 38 | 29 | 3.1 |
| 166 | AGG325633 | SA | 0.89 | 0.52 | 0.35 | 0.27 | 1.24 | 0.79 | 14 | 38 | 28 | 3.7 |
| 167 | AGG325634 | SA | 0.60 | 0.39 | 0.25 | 0.17 | 0.85 | 0.56 | 23 | 42 | 26 | 2.9 |
| 168 | AGG325635 | SA | 0.50 | 0.29 | 0.21 | 0.14 | 0.71 | 0.42 | 15 | 44 | 27 | 4.2 |
| 169 | AGG325636 | SA | 0.37 | 0.22 | 0.13 | 0.09 | 0.50 | 0.32 | 10 | 39 | 30 | 4.1 |
| 170 | AGG325637 | SA | 0.81 | 0.57 | 0.37 | 0.23 | 1.18 | 0.81 | 14 | 36 | 27 | 3.4 |
| 171 | AGG325638 | SA | 0.59 | 0.28 | 0.22 | 0.10 | 0.81 | 0.38 | 11 | 35 | 27 | 3.1 |
| 172 | AGG325639 | SA | 0.57 | 0.44 | 0.27 | 0.19 | 0.84 | 0.63 | 12 | 40 | 30 | 2.1 |
| 173 | AGG325640 | SA | 0.72 | 0.30 | 0.32 | 0.11 | 1.04 | 0.41 | 8 | 36 | 30 | 3.8 |
| 174 | AGG325641 | SA | 0.47 | 0.36 | 0.19 | 0.14 | 0.66 | 0.49 | 8 | 37 | 28 | 2.9 |
| 175 | AGG325642 | SA | 0.74 | 0.58 | 0.36 | 0.23 | 1.11 | 0.82 | 15 | 38 | 23 | 5.2 |
| 176 | AGG325643 | SA | 1.04 | 0.48 | 0.43 | 0.23 | 1.47 | 0.71 | 14 | 37 | 26 | 4.8 |
| 177 | AGG325644 | SA | 0.44 | 0.25 | 0.26 | 0.15 | 0.70 | 0.40 | 13 | 40 | 26 | 3.4 |
| 178 | AGG325645 | SA | 0.53 | 0.28 | 0.31 | 0.12 | 0.84 | 0.40 | 7 | 36 | 25 | 3.5 |
| 179 | AGG325646 | SA | 0.75 | 0.41 | 0.33 | 0.18 | 1.08 | 0.59 | 11 | 33 | 26 | 3.6 |
| 180 | AGG325647 | SA | 0.69 | 0.50 | 0.21 | 0.16 | 0.90 | 0.65 | 13 | 37 | 26 | 3.7 |
| 181 | AGG325648 | SA | 0.62 | 0.44 | 0.31 | 0.13 | 0.92 | 0.57 | 9 | 38 | 26 | 3.4 |
| 182 | AGG325649 | UK | 0.58 | 0.32 | 0.30 | 0.11 | 0.87 | 0.43 | 11 | 36 | 25 | 3.0 |
| 183 | AGG325650 | SA | 0.64 | 0.57 | 0.41 | 0.22 | 1.04 | 0.79 | 13 | 37 | 24 | 4.2 |
| 184 | AGG325651 | SA | 1.28 | 0.65 | 0.58 | 0.30 | 1.86 | 0.95 | 11 | 34 | 26 | 3.0 |
| 185 | AGG325652 | SA | 0.95 | 0.58 | 0.49 | 0.26 | 1.45 | 0.84 | 10 | 39 | 36 | 3.2 |
| 186 | AGG325653 | SA | 0.48 | 0.35 | 0.25 | 0.16 | 0.73 | 0.51 | 9 | 35 | 29 | 4.1 |
| 187 | AGG325654 | SA | 0.51 | 0.38 | 0.24 | 0.12 | 0.76 | 0.50 | 10 | 37 | 29 | 3.2 |
| 188 | AGG325655 | SA | 0.70 | 0.24 | 0.40 | 0.14 | 1.09 | 0.38 | 10 | 40 | 22 | 3.6 |
| 189 | AGG325656 | SA | 0.65 | 0.35 | 0.46 | 0.14 | 1.12 | 0.49 | 10 | 35 | 25 | 3.2 |
| 190 | AGG325657 | SA | 0.74 | 0.45 | 0.26 | 0.17 | 1.00 | 0.62 | 11 | 35 | 31 | 3.1 |
| 191 | AGG325658 | SA | 1.04 | 0.44 | 0.44 | 0.18 | 1.48 | 0.62 | 11 | 42 | 27 | 3.7 |
| 192 | AGG325659 | SA | 0.48 | 0.23 | 0.26 | 0.07 | 0.75 | 0.30 | 7 | 33 | 26 | 2.7 |
| 193 | AGG325660 | SA | 0.63 | 0.22 | 0.33 | 0.12 | 0.96 | 0.34 | 8 | 33 | 26 | 3.3 |
| 194 | AGG325662 | SA | 0.65 | 0.38 | 0.31 | 0.12 | 0.96 | 0.49 | 12 | 37 | 24 | 2.9 |
| 195 | AGG325663 | SA | 0.49 | 0.32 | 0.27 | 0.16 | 0.76 | 0.49 | 7 | 40 | 23 | 3.3 |
| 196 | AGG325664 | SA | 0.57 | 0.32 | 0.23 | 0.11 | 0.80 | 0.42 | 8 | 38 | 26 | 2.8 |
| 197 | AGG325665 | SA | 0.53 | 0.30 | 0.28 | 0.14 | 0.81 | 0.44 | 7 | 36 | 28 | 5.4 |
| 198 | AGG325666 | SA | 0.66 | 0.37 | 0.34 | 0.15 | 1.00 | 0.53 | 15 | 37 | 28 | 3.0 |
| 199 | AGG325667 | SA | 0.72 | 0.37 | 0.24 | 0.13 | 0.95 | 0.50 | 7 | 40 | 29 | 3.0 |
| 200 | AGG325668 | SA | 0.61 | 0.35 | 0.24 | 0.14 | 0.85 | 0.48 | 4 | 34 | 26 | 4.9 |
| 201 | AGG325669 | SA | 0.66 | 0.27 | 0.32 | 0.15 | 0.97 | 0.42 | 11 | 39 | 27 | 3.6 |
| 202 | AGG325670 | SA | 0.57 | 0.29 | 0.25 | 0.13 | 0.83 | 0.42 | 8 | 37 | 26 | 3.8 |
| 203 | AGG325671 | SA | 0.22 | 0.15 | 0.16 | 0.09 | 0.38 | 0.24 | 9 | 41 | 30 | 4.5 |
| 204 | AGG325672 | SA | 0.59 | 0.35 | 0.25 | 0.12 | 0.84 | 0.47 | 8 | 37 | 30 | 4.4 |
| 205 | AGG325673 | SA | 1.25 | 0.64 | 0.60 | 0.26 | 1.85 | 0.90 | 17 | 42 | 23 | 3.4 |
| 206 | AGG325674 | SA | 1.02 | 0.49 | 0.51 | 0.28 | 1.53 | 0.77 | 19 | 40 | 27 | 4.7 |
| 207 | AGG325675 | SA | 0.66 | 0.31 | 0.47 | 0.15 | 1.13 | 0.46 | 13 | 38 | 17 | 3.1 |
| 208 | AGG325676 | SA | 0.96 | 0.55 | 0.55 | 0.32 | 1.50 | 0.87 | 16 | 40 | 16 | 3.3 |
| 209 | AGG325677 | SA | 1.08 | 0.46 | 0.57 | 0.20 | 1.65 | 0.66 | 16 | 38 | 23 | 4.0 |
| 210 | AGG325678 | SA | 0.67 | 0.50 | 0.31 | 0.23 | 0.98 | 0.73 | 15 | 40 | 17 | 5.7 |
| 211 | AGG325679 | SA | 1.11 | 0.39 | 0.53 | 0.22 | 1.65 | 0.61 | 16 | 40 | 24 | 3.1 |
| 212 | AGG325680 | SA | 0.50 | 0.34 | 0.25 | 0.14 | 0.75 | 0.48 | 13 | 40 | 28 | 3.2 |
| 213 | AGG325681 | SA | 0.72 | 0.49 | 0.38 | 0.26 | 1.10 | 0.76 | 11 | 41 | 22 | 3.1 |
| 214 | AGG325682 | SA | 0.50 | 0.37 | 0.30 | 0.20 | 0.80 | 0.57 | 11 | 32 | 25 | 3.4 |
| 215 | AGG325683 | SWA | 1.32 | 0.42 | 0.69 | 0.29 | 2.02 | 0.71 | 19 | 34 | 21 | 3.0 |
| 216 | AGG325684 | SA | 1.77 | 0.71 | 1.07 | 0.39 | 2.84 | 1.10 | 16 | 37 | 28 | 4.0 |
| 217 | AGG325685 | SA | 0.91 | 0.54 | 0.45 | 0.25 | 1.35 | 0.80 | 12 | 38 | 26 | 3.2 |
| 218 | AGG325686 | SA | 1.05 | 0.65 | 0.56 | 0.28 | 1.60 | 0.93 | 19 | 36 | 24 | 3.5 |
| 219 | AGG325687 | SA | 0.66 | 0.32 | 0.28 | 0.21 | 0.93 | 0.52 | 12 | 38 | 26 | 3.4 |
| 220 | AGG325688 | SWA | 0.92 | 0.34 | 0.43 | 0.17 | 1.35 | 0.51 | 10 | 39 | 20 | 6.8 |
| 221 | AGG325689 | SA | 1.22 | 0.48 | 0.48 | 0.22 | 1.70 | 0.70 | 13 | 38 | 24 | 4.3 |
| 222 | AGG325690 | SA | 0.77 | 0.46 | 0.47 | 0.24 | 1.24 | 0.70 | 11 | 38 | 20 | 2.8 |
| 223 | AGG325691 | SA | 0.61 | 0.47 | 0.32 | 0.25 | 0.93 | 0.73 | 16 | 37 | 28 | 3.5 |
| 224 | AGG325692 | SA | 0.77 | 0.39 | 0.45 | 0.19 | 1.22 | 0.58 | 14 | 39 | 23 | 3.6 |
| 225 | AGG325693 | SWA | 0.72 | 0.40 | 0.52 | 0.23 | 1.24 | 0.63 | 13 | 40 | 25 | 3.9 |
| 226 | AGG325694 | SWA | 0.97 | 0.55 | 0.59 | 0.31 | 1.56 | 0.86 | 18 | 34 | 25 | 3.6 |
| 227 | AGG325695 | SA | 1.18 | 0.72 | 0.73 | 0.46 | 1.91 | 1.17 | 15 | 38 | 22 | 3.7 |
| 228 | AGG325696 | SA | 0.80 | 0.43 | 0.38 | 0.26 | 1.18 | 0.69 | 11 | 38 | 26 | 3.5 |
| 229 | AGG325697 | SA | 1.09 | 0.58 | 0.66 | 0.29 | 1.75 | 0.88 | 14 | 38 | 24 | 4.0 |
| 230 | AGG325698 | SA | 0.72 | 0.62 | 0.38 | 0.35 | 1.10 | 0.97 | 20 | 38 | 23 | 3.4 |
| 231 | AGG325699 | SA | 1.18 | 0.51 | 0.65 | 0.33 | 1.83 | 0.83 | 15 | 42 | 21 | 3.1 |
| 232 | AGG325700 | SA | 0.84 | 0.40 | 0.48 | 0.15 | 1.32 | 0.55 | 10 | 38 | 27 | 2.5 |
| 233 | AGG325701 | SA | 0.79 | 0.37 | 0.45 | 0.19 | 1.23 | 0.56 | 19 | 38 | 26 | 4.2 |
| 234 | AGG325702 | SA | 0.52 | 0.41 | 0.28 | 0.22 | 0.79 | 0.64 | 16 | 37 | 18 | 3.4 |
| 235 | AGG325703 | SA | 1.10 | 0.46 | 0.55 | 0.25 | 1.65 | 0.71 | 15 | 37 | 25 | 2.7 |
| 236 | AGG325704 | OP | 0.86 | 0.71 | 0.47 | 0.36 | 1.32 | 1.07 | 17 | 38 | 20 | 5.4 |
| 237 | AGG325705 | SA | 1.03 | 0.47 | 0.49 | 0.23 | 1.52 | 0.70 | 12 | 36 | 24 | 4.0 |
| 238 | AGG325706 | SA | 1.08 | 0.41 | 0.67 | 0.21 | 1.75 | 0.62 | 17 | 37 | 25 | 4.0 |
| 239 | AGG325707 | SA | 0.97 | 0.60 | 0.53 | 0.29 | 1.51 | 0.89 | 13 | 40 | 22 | 3.7 |
| 240 | AGG325708 | SA | 0.70 | 0.38 | 0.27 | 0.14 | 0.97 | 0.52 | 12 | 37 | 29 | 3.6 |
| 241 | AGG325709 | SA | 0.95 | 0.32 | 0.49 | 0.20 | 1.44 | 0.52 | 11 | 36 | 24 | 3.4 |
| 242 | AGG325710 | SA | 0.79 | 0.35 | 0.32 | 0.15 | 1.12 | 0.50 | 13 | 35 | 18 | 3.0 |
| 243 | AGG325711 | UK | 0.71 | 0.30 | 0.37 | 0.14 | 1.07 | 0.44 | 15 | 38 | 22 | 3.1 |
| 244 | AGG325712 | SA | 0.44 | 0.43 | 0.32 | 0.20 | 0.76 | 0.63 | 10 | 39 | 25 | 2.8 |
| 245 | AGG325713 | SWA | 1.02 | 0.38 | 0.60 | 0.17 | 1.61 | 0.55 | 14 | 39 | 23 | 3.3 |
| 246 | AGG325714 | EUR | 0.73 | 0.39 | 0.45 | 0.26 | 1.18 | 0.65 | 14 | 38 | 23 | 3.1 |
| 247 | AGG325715 | SWA | 0.58 | 0.32 | 0.39 | 0.18 | 0.97 | 0.51 | 14 | 35 | 23 | 3.8 |
| 248 | AGG325716 | SA | 1.11 | 0.47 | 0.64 | 0.26 | 1.75 | 0.73 | 10 | 37 | 27 | 3.0 |
| 249 | AGG325717 | SWA | 1.03 | 0.52 | 0.62 | 0.32 | 1.65 | 0.85 | 9 | 36 | 26 | 3.9 |
| 250 | AGG325718 | SWA | 0.98 | 0.38 | 0.54 | 0.23 | 1.53 | 0.60 | 22 | 37 | 25 | 2.9 |
| 251 | AGG325719 | SWA | 0.91 | 0.28 | 0.62 | 0.19 | 1.52 | 0.46 | 16 | 36 | 25 | 4.2 |
| 252 | AGG325720 | SA | 0.75 | 0.28 | 0.53 | 0.18 | 1.28 | 0.46 | 9 | 38 | 25 | 3.2 |
| 253 | AGG325721 | SA | 1.44 | 0.48 | 0.81 | 0.30 | 2.25 | 0.78 | 14 | 40 | 24 | 3.8 |
| 254 | AGG325722 | SA | 1.24 | 0.34 | 0.65 | 0.15 | 1.90 | 0.49 | 12 | 38 | 25 | 3.4 |
| 255 | AGG325723 | SWA | 0.93 | 0.38 | 0.67 | 0.22 | 1.60 | 0.60 | 14 | 38 | 30 | 4.0 |
| 256 | AGG325724 | SWA | 0.67 | 0.33 | 0.44 | 0.21 | 1.11 | 0.54 | 11 | 36 | 23 | 3.4 |
| 257 | AGG325725 | SWA | 0.74 | 0.40 | 0.55 | 0.26 | 1.29 | 0.66 | 11 | 36 | 25 | 5.3 |
| 258 | AGG325726 | SWA | 0.98 | 0.48 | 0.80 | 0.34 | 1.78 | 0.82 | 21 | 39 | 26 | 3.3 |
| 259 | AGG325727 | SWA | 0.73 | 0.24 | 0.49 | 0.15 | 1.23 | 0.38 | 12 | 37 | 25 | 5.3 |
| 260 | AGG325728 | SWA | 0.84 | 0.48 | 0.60 | 0.33 | 1.44 | 0.82 | 15 | 37 | 24 | 2.9 |
| 261 | AGG325729 | SWA | 0.87 | 0.53 | 0.62 | 0.30 | 1.49 | 0.83 | 16 | 36 | 28 | 3.2 |
| 262 | AGG325730 | SWA | 0.61 | 0.54 | 0.37 | 0.32 | 0.98 | 0.86 | 9 | 39 | 26 | 3.8 |
| 263 | AGG325731 | SWA | 0.94 | 0.50 | 0.48 | 0.25 | 1.41 | 0.75 | 15 | 38 | 21 | 3.5 |
| 264 | AGG325732 | SA | 1.08 | 0.99 | 0.72 | 0.51 | 1.80 | 1.50 | 13 | 36 | 25 | 2.7 |
| 265 | AGG325733 | SA | 1.12 | 0.60 | 0.59 | 0.25 | 1.71 | 0.85 | 23 | 40 | 22 | 2.6 |
| 266 | AGG325734 | SA | 1.62 | 0.81 | 0.89 | 0.45 | 2.51 | 1.26 | 19 | 42 | 26 | 3.1 |
| 267 | AGG325735 | SA | 1.01 | 0.43 | 0.41 | 0.21 | 1.42 | 0.64 | 15 | 36 | 25 | 3.1 |
| 268 | AGG325736 | SA | 0.80 | 0.43 | 0.43 | 0.25 | 1.23 | 0.68 | 16 | 41 | 28 | 2.9 |
| 269 | AGG325737 | SA | 0.69 | 0.28 | 0.41 | 0.16 | 1.10 | 0.44 | 11 | 38 | 23 | 4.0 |
| 270 | AGG325738 | SA | 0.60 | 0.57 | 0.29 | 0.27 | 0.89 | 0.84 | 18 | 33 | 22 | 7.5 |
| 271 | AGG325739 | SA | 0.81 | 0.34 | 0.55 | 0.18 | 1.36 | 0.53 | 14 | 37 | 26 | 3.4 |
| 272 | AGG325740 | SA | 0.72 | 0.45 | 0.26 | 0.19 | 0.98 | 0.64 | 12 | 36 | 24 | 6.9 |
| 273 | AGG325741 | SA | 0.67 | 0.39 | 0.40 | 0.19 | 1.07 | 0.59 | 15 | 35 | 22 | 3.3 |
| 274 | AGG325742 | SA | 0.91 | 0.38 | 0.39 | 0.20 | 1.29 | 0.57 | 19 | 41 | 26 | 3.8 |
| 275 | AGG325743 | SWA | 0.99 | 0.60 | 0.50 | 0.25 | 1.50 | 0.84 | 16 | 38 | 15 | 4.0 |
| 276 | AGG325744 | SWA | 0.92 | 0.47 | 0.44 | 0.19 | 1.35 | 0.65 | 9 | 38 | 24 | 3.3 |
| 277 | AGG325745 | SWA | 0.65 | 0.45 | 0.25 | 0.20 | 0.90 | 0.65 | 15 | 38 | 21 | 3.0 |
| 278 | AGG325746 | SWA | 1.01 | 0.47 | 0.45 | 0.23 | 1.46 | 0.70 | 12 | 40 | 20 | 3.7 |
| 279 | AGG325747 | SWA | 0.75 | 0.42 | 0.44 | 0.19 | 1.19 | 0.61 | 12 | 38 | 20 | 3.8 |
| 280 | AGG325748 | SWA | 1.17 | 0.72 | 0.62 | 0.26 | 1.78 | 0.99 | 19 | 38 | 27 | 3.4 |
| 281 | AGG325749 | SWA | 1.33 | 0.65 | 0.72 | 0.22 | 2.05 | 0.87 | 20 | 40 | 22 | 3.4 |
| 282 | AGG325750 | SWA | 0.44 | 0.38 | 0.20 | 0.18 | 0.64 | 0.56 | 14 | 35 | 22 | 3.4 |
| 283 | AGG325751 | SWA | 0.82 | 0.37 | 0.46 | 0.25 | 1.28 | 0.62 | 18 | 38 | 29 | 3.4 |
| 284 | AGG325752 | SWA | 0.66 | 0.49 | 0.37 | 0.25 | 1.03 | 0.74 | 14 | 37 | 26 | 2.5 |
| 285 | AGG325753 | SWA | 1.12 | 0.49 | 0.48 | 0.29 | 1.60 | 0.79 | 18 | 40 | 26 | 3.3 |
| 286 | AGG325754 | SWA | 0.78 | 0.48 | 0.35 | 0.26 | 1.13 | 0.74 | 12 | 38 | 16 | 3.3 |
| 287 | AGG325755 | SA | 0.53 | 0.43 | 0.31 | 0.19 | 0.83 | 0.63 | 16 | 40 | 18 | 3.4 |
| 288 | AGG325756 | SA | 0.76 | 0.48 | 0.40 | 0.27 | 1.17 | 0.75 | 16 | 40 | 27 | 3.6 |
| 289 | AGG325758 | MA | 1.57 | 0.91 | 0.86 | 0.59 | 2.43 | 1.50 | 15 | 40 | 23 | 3.2 |
| 290 | AGG325759 | UK | 0.95 | 0.52 | 0.56 | 0.30 | 1.51 | 0.82 | 18 | 40 | 24 | 3.3 |
| 291 | AGG325760 | SA | 1.01 | 0.57 | 0.53 | 0.27 | 1.54 | 0.84 | 16 | 36 | 28 | 3.3 |
| 292 | AGG325761 | AFR | 1.52 | 0.94 | 0.83 | 0.42 | 2.35 | 1.36 | 16 | 39 | 25 | 3.8 |
